# GCE: A Framework for Interpretable Nonlinear Hazard Modeling in Cardiac Sarcoma Survival Using SEER Data

**DOI:** 10.3390/bioengineering13080891

**Published:** 2026-08-01

**Authors:** Muhammad Shoaib Kareem, Madiha Amjad, Saba Aslam, Abdur Rasool, Mutiullah Jamil, Hazrat Ali

**Affiliations:** 1 Department of Information Technology, Khawaja Fareed University of Engineering and Technology, Rahim Yar Khan 64200, Pakistan or inft201700004@kfueit.edu.pk (M.S.K.); madihaamjad@cuilahore.edu.pk (M.A.); mutiullah@kfueit.edu.pk (M.J.); 2 Department of Computer Science, COMSATS University Islamabad, Lahore Campus, Lahore 54000, Pakistan; 3 Shenzhen Institutes of Advanced Technology, Chinese Academy of Sciences, Shenzhen 518055, China; aslam@siat.ac.cn; 4 Department of Information and Computer Sciences, University of Hawaii at Manoa, Honolulu, HI 96822, USA; 5 School of Computing Data and Mathematical Sciences, University of Stirling, Stirling FK9 4LA, UK

**Keywords:** cardiac sarcoma, survival analysis, cox proportional hazards, single-step static covariates, nonlinear risk modeling, post hoc SHAP association, SEER database

## Abstract

Cardiac sarcoma is a rare aggressive malignancy for which survival prediction is limited by small cohorts, censoring, nonlinear prognostic effects, and incomplete interpretability. We developed the GRU–CoxPH Ensemble (GCE), a weighted late-fusion survival framework combining a single-step GRU static-covariate learner with Cox proportional hazards (CoxPHs). Using SEER Research Plus data, 727 eligible patients were identified from 41 source variables, and 27 raw SEER predictors were retained after leakage exclusion and Cox-LASSO/Random Survival Forest screening. Outcome, follow-up, cause-of-death, identifier, and endpoint-derived fields were removed before preprocessing; imputation, encoding, scaling, feature selection, tuning, and ensemble-weight selection were performed within training folds only. In leakage-free 10-fold out-of-fold evaluation, the GCE achieved high cohort-specific internal discrimination that is likely optimistic and requires external validation (mean C-index =0.9198; IBS = 0.03641), whereas CoxPH showed lower discrimination (C-index = 0.8605) but better IBS calibration (0.03378). Thus, the GCE is better for discrimination, under internal validation, and is not calibration-superior. SHAP provided post hoc descriptive association summaries for the final ensemble risk score. These internal results support the GCE only as a research framework for cardiac sarcoma survival-risk modeling; external validation and recalibration are required before any patient-level clinical consideration.

## 1. Introduction

**Figure 1:**
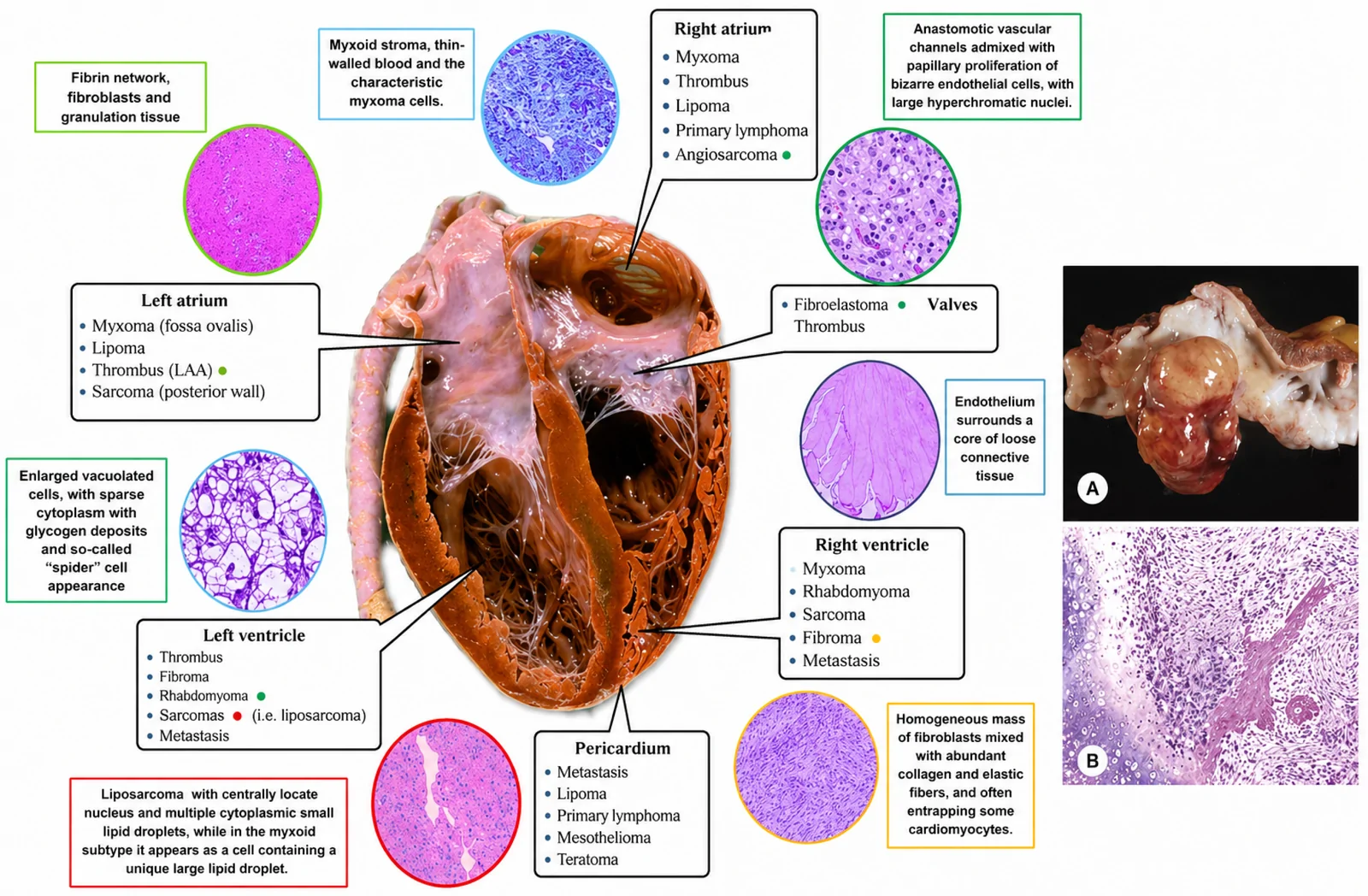
Representative images of cardiac angiosarcoma. **Left**: macroscopic appearance showing a nodular, hemorrhagic lesion in the heart. **Right**: microscopic features showing irregular vascular spaces lined by atypical endothelial cells. Adapted from [4] under the Creative Commons Attribution 4.0 International (CC BY 4.0) license; no endorsement by the original source is implied.

Cardiac sarcoma is a rare and aggressive malignancy with poor prognosis, often with median survival ranging from seven months to three years after diagnosis [1,2]. Prognostication is difficult because cases are sparse, histology is heterogeneous, and treatment effects are confounded by local invasion, metastasis, censoring, and non-cancer mortality [2,3]. Reliable risk estimation is clinically important because survival varies across tumor stage, treatment exposure, and patient factors, yet individual institutions rarely accumulate cohorts large enough for stable model development. Figure 1 shows representative cardiac angiosarcoma morphology [4].

Population registries such as SEER provide one of the few data sources large enough for cardiac sarcoma survival modeling [5]. However, SEER-based prediction remains challenging because registry variables are static, rare-disease cohorts are small, and Cox proportional hazards (CoxPHs) models may miss nonlinear covariate-risk associations [3,6,7,8]. Machine-learning and deep survival models can capture nonlinear effects, but internal retrospective results should not be interpreted as patient-management recommendations without external validation [9,10,11,12,13,14,15]. SEER-based modeling also requires strict separation of registry-landmark predictors from survival labels and follow-up fields so that performance reflects prognostic signal rather than target leakage.

Accordingly, this study evaluates the GRU–CoxPH Ensemble (GCE), a weighted late-fusion survival framework that combines a single-step GRU static-covariate learner with CoxPH statistical anchoring. Because SEER contains one registry-landmark record per patient rather than longitudinal covariate sequences, the GRU is used only as a nonlinear static learner, not as a model of temporal disease evolution. A parameter-matched MLP comparator is included to contextualize whether nonlinear static learning, rather than recurrence over patient trajectories, explains model behavior. Ensemble weights are selected by fold-isolated inner-validation grid search (Section 3.3 and Section 3.5). The objective is to test whether the GCE improves discrimination (C-index) while jointly reporting calibration (IBS); calibration superiority is not claimed when a simpler model has a lower IBS.

The main contributions are the following:

- A weighted late-fusion GRU–CoxPH survival framework for nonlinear risk modeling. Because SEER lacks longitudinal covariate sequences, the GRU branch is interpreted as a single-step static neural learner.
- Post hoc SHAP association summaries and CoxPH anchoring to improve transparency of nonlinear risk scores based on registry-landmark covariates.
- Leakage-controlled rare-disease validation using Cox-LASSO/RSF feature screening, 10-fold out-of-fold evaluation, bootstrap uncertainty, and outcome-permutation negative controls in a SEER cohort of 727 patients.

The GCE is evaluated as an internally validated survival-risk modeling framework for rare cardiac sarcoma, with cautious interpretation pending external validation. The high internal C-index should be considered cohort-specific and likely optimistic because rare-cancer cohorts can be relatively homogeneous and may contain strong registry-landmark predictors. This paper is structured as follows: Section 1 provides the introduction. Section 2 presents the literature review. Section 3 details the methodology, including data, variables, and model development. Section 4 presents the results. Section 5 reports the discussion and limitations. Section 6 provides the conclusion.

## 2. Literature Review

### 2.1. Cancer Survival Prediction Using AI

The literature review was refocused on studies most relevant to primary cardiac sarcoma survival prediction: (i) population-based cardiac tumor or cardiac sarcoma outcome studies, (ii) rare sarcoma or rare bone-tumor survival prediction studies, and (iii) modern survival-learning methods such as DeepSurv, MTLR, DeepHit, RSF, and hybrid statistical–deep models. SEER provides population-level incidence, treatment, and survival information for rare-cancer analyses [16]. Cardiac sarcoma, cardiac angiosarcoma, and primary cardiac lymphoma studies based on SEER or NCDB remain largely descriptive or regression-based rather than predictive [2,6,17,18]. Recent rare-tumor and registry-based prediction studies provide closer methodological context than broad comparisons with common malignancies. Machine-learning models have been developed for chordoma survival [19], deep learning has been applied to chondrosarcoma, osteosarcoma, gastrointestinal stromal tumor, lung cancer, and glioblastoma survival prediction [9,20,21,22,23], and RSF has been used for spinal chordoma prognosis [24]. Modern DeepSurv, multimodal survival, ensemble, and hybrid CoxPH–DeepHit frameworks show how nonlinear models can complement Cox-based methods, but most are non-cardiac and are used here only as methodological context [25,26,27,28,29,30]. Table 1 summarizes the most relevant cardiac-tumor, rare-cancer, and survival-learning studies used to position the proposed framework.

### 2.2. Challenges in SEER-Based Prognostic Modeling

SEER studies are limited by retrospective bias, incomplete variables (e.g., comorbidities, treatment dose, genomic markers, lifestyle factors), U.S.-based registry coverage, and possible rare-event under-ascertainment [6,16,31,32,33,34]. In sparse rare-cancer cohorts, CoxPH, logistic regression, and Kaplan–Meier summaries may miss nonlinear interactions, whereas ML and deep survival models often lack calibration reporting, external validation, or transparent interpretation [9,19,21,34]. Table 1 therefore motivates GCE as an internal survival-modeling approach without direct superiority claims over heterogeneous external studies. These challenges are amplified in cardiac sarcoma because the disease is rare, the eligible cohort is small, and patient characteristics vary by age, histology, tumor extension, treatment receipt, and follow-up duration. Censoring reduces directly observed event information for model fitting, and missing clinical detail limits adjustment for treatment intensity, comorbidity, and disease burden [24,26,27,35]. SEER does not provide repeated longitudinal measurements or integrated cardiac imaging, pathology images, genomics, or treatment-dose trajectories for this cohort. Consequently, model generalization depends on leakage-free validation, strict exclusion of outcome and follow-up fields, and cautious interpretation of internal cross-validation results before external application [36,37,38,39].

## 3. Materials and Methods

### 3.1. Data Collection and Patient Selection

This retrospective cohort study used SEER Research Plus Data, 21 Registries, November 2024 submission (excluding Illinois), accessed through SEER*Stat version 8.4.1 [16]. Primary cardiac sarcoma cases diagnosed from 2000 to 2022 were identified using ICD-O-3, with primary site C38.0 (heart), malignant behavior (/3), and cardiac sarcoma histology groups represented in the export. From 16,057,864 initial records, 727 eligible patients remained after retaining malignant primary records, resolving duplicate patient records by earliest eligible diagnosis, and requiring valid survival time and vital-status information; complete case-flow counts are provided in Supplementary Figure S3, and extraction rules are provided in Supplementary Table S11.

To prevent target leakage, modeling starts from the raw SEER export. Survival months is used only as the survival-time label *T*, Vital status recode is used only to define the event indicator δ, and the outcome, follow-up, cause-of-death, endpoint-derived, future-derived, non-applicable, and identifier fields are removed before imputation, encoding, scaling, feature selection, model fitting, SHAP analysis, or figure generation. Table 2 defines each variable role, and explicit audits confirmed that no outcome-like or follow-up-like column remained in the predictor matrix.

The prediction timestamp is a post-diagnostic SEER registry landmark after diagnostic confirmation and initial-treatment abstraction. Demographic and tumor variables are covariates, whereas the surgery, chemotherapy, and reason-for-no-surgery fields are interpreted only as landmark covariates available by registry abstraction; lifetime tumor-count fields are excluded as potentially future-derived. The complete timestamp and retained-variable audit is provided in Supplementary Table S8.

The primary endpoint is overall survival, defined as time from diagnosis to death from any cause. Patients alive at last follow-up are censored; cardiovascular, treatment-related, and other non-sarcoma deaths are counted as events rather than competing censored events. Vital status recode was mapped as 5 = death/event and 0 = alive/censored, and zero-month records, if present, were retained as tied survival times. Cause-specific or Fine–Gray analyses are reserved for future endpoint-specific sensitivity analyses. Additional endpoint details are provided in Supplementary Table S11.

The analysis summarizes cohort endpoints before model fitting, including cohort size, uncensored deaths/events, censoring, median survival, reverse Kaplan–Meier median follow-up, and event/censoring distribution within each validation fold. Baseline clinical characteristics are summarized for the final predictor set using the median and interquartile range for numeric variables and the frequency with percentage for categorical variables. These summaries are generated before model training and are not used as predictors.

### 3.2. Feature Selection

A total of 41 SEER source fields were retrieved, including candidate predictors and endpoint fields required to define (T,δ). Eligible predictors were restricted to variables available by the post-diagnostic SEER registry landmark, and the outcome, follow-up, identifier, future-derived, and non-applicable fields were removed before preprocessing or feature selection. Variables with excessive missingness (>20%) were excluded a priori; the complete data dictionary, exclusion rationale, timing, and encoding plan are provided in Supplementary Table S8. Feature selection used survival-aware Cox-LASSO and RSF screening [9,29,40,41]. Cox-LASSO uses penalized partial likelihood with survival time and censoring status, whereas RSF contributes nonlinear variable-importance ranking. The final GCE input is the 27-variable Cox-LASSO–RSF union/consensus raw feature set; Cox-LASSO-only, RSF-only, and PCA-transformed representations are comparator or sensitivity analyses, not final GCE inputs.

(1) $$\min_{\beta }[-\frac{1}{n}\sum_{i=1}^{n}\delta_{i}(x_{i}^{T}\beta -\log\sum_{j\in R(t_{i})}\exp\left(x_{j}^{T}\beta \right))+\lambda {∥\beta ∥}_{1}]$$

where λ is optimized within the inner training data of each outer fold to balance sparsity and survival-discrimination performance; the held-out outer fold is not used for penalty selection.

Fold-wise selection stability was quantified as follows:

(2) $$SF_{j}=\frac{1}{K}\sum_{k=1}^{K}I_{j}^{\left(k\right)}$$

where $SF_{j}$ is the proportion of folds in which feature *j* is selected. The retained variables were grouped as demographic, tumor, and treatment-landmark fields for interpretability. The term “27 features” refers to 27 raw SEER fields before one-hot encoding, not encoded design-matrix columns. CoxPH, GRU, the parameter-matched MLP, and the GCE receive the same raw-field definition before fold-specific preprocessing and Cox-LASSO masking. Cox-LASSO penalties, selected-feature counts, raw-feature frequencies, and encoded columns are reported in Supplementary Table S9.

**Figure 2:**
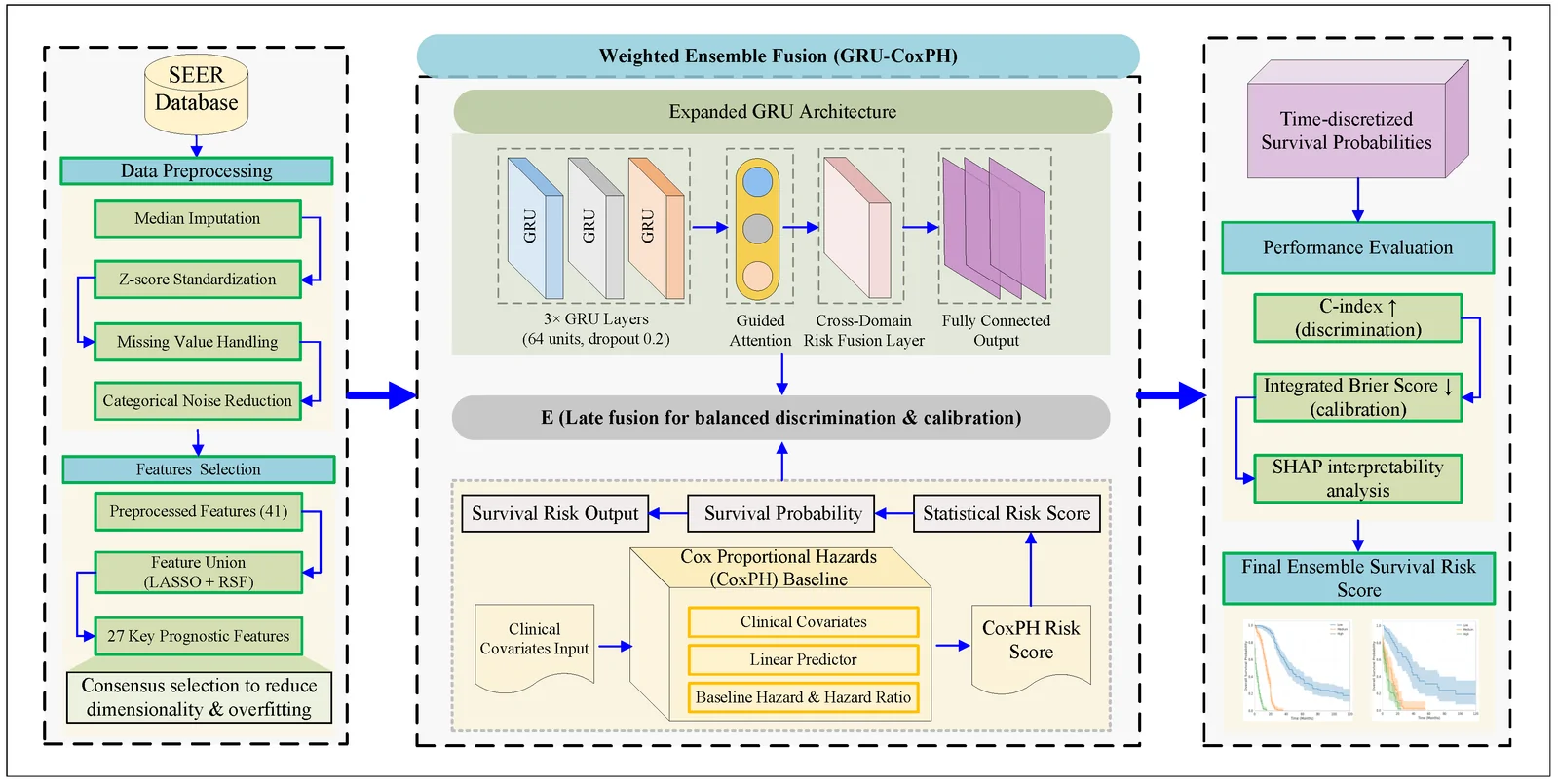
The Schematic workflow of the GCE framework, including SEER data extraction and cohort curation, feature selection, parallel training of survival learners under 10-fold CV, weighted late fusion, discrimination assessment using the C-index, calibration assessment using the IBS, and post hoc SHAP analysis. The neural branch uses a single-step static covariate representation, and the fusion block denotes convex averaging of standardized CoxPH and GRU risk scores rather than attention, dynamic gating, or longitudinal recurrence. The interpretation distinguishes internal discrimination from calibration and does not present the GCE as calibration-superior to CoxPH when CoxPH has the lower IBS.

Feature selection was performed only within training folds. Table 3 summarizes the unified workflow, and Figure 2 summarizes the GCE workflow.

### 3.3. Proposed GCE Framework

The GCE is a weighted late-fusion framework that combines the nonlinear representation capacity of a GRU with CoxPH statistical anchoring. Because SEER provides static registry-landmark covariates rather than longitudinal sequences, the GRU is interpreted as a single-step nonlinear static learner; no attention module, dynamic gate, time-varying fusion, longitudinal recurrence, or disease-trajectory model is claimed. A parameter-matched MLP comparator was trained on the same folds and encoded inputs to assess whether a feed-forward static learner provides similar nonlinear information. The workflow is shown in Figure 2.

After training the CoxPH and GRU components, the patient-specific ensemble risk score $E_{i}$ is as follows:

(3) $$E_{i}=w_{\mathrm{CoxPH}}\cdot S_{\mathrm{CoxPH},i}+w_{\mathrm{GRU}}\cdot S_{\mathrm{GRU},i}$$

where $S_{\mathrm{CoxPH},i}$ is the CoxPH log-risk, $S_{\mathrm{GRU},i}$ is the GRU risk output, and $w_{\mathrm{CoxPH}}+w_{\mathrm{GRU}}=1$. Component risks are standardized using training-fold means and standard deviations:

(4) $${\tilde{S}}_{m,i}=\frac{S_{m,i}-\mu_{m}}{\sigma_{m}},\;E_{i}=\sum_{m\in \{\mathrm{CoxPH},\mathrm{GRU}\}}w_{m}\cdot {\tilde{S}}_{m,i}$$

where $\mu_{m}$ and $\sigma_{m}$ are computed from training data for model *m*. Within each outer training fold, CoxPH and GRU are fitted on inner-training data, component risks are standardized using inner-training parameters only, candidate GRU weights {0.0,0.1,…,1.0} are evaluated on the inner validation split by C-index, and the selected convex weighting is then applied to the untouched outer validation fold. The final reported summary weighting is $w_{\mathrm{CoxPH}}=0.2$ and $w_{\mathrm{GRU}}=0.8$.

Fusion is performed on risk scores, not survival probabilities. The IBS is computed from model-derived survival estimates: fitted CoxPH survival curves for CoxPH and training-fold Breslow baseline survival estimates for neural or fused risk models. The C-index is Harrell’s concordance for right-censored data, with higher risk scores denoting higher hazard; score direction is fixed by model semantics. Table 4 lists the model hyperparameters, training settings, and evaluation configuration.

The neural component applies three stacked GRU layers (64 hidden units each, dropout = 0.2) to a single-step representation of the registry-landmark covariate vector; it is a nonlinear static learner and is not interpreted as learning longitudinal dependencies. The MLP comparator uses the same selected encoded inputs as a feed-forward reference for static nonlinear learning. Let $h_{i}$ denote the final hidden representation for patient *i*. The neural risk score is as follows:

(5) $$S_{\mathrm{GRU},i}=g_{\theta }\left(h_{i}\right)$$

This score models nonlinear associations among registry-landmark covariates; repeated observations or longitudinal trajectories are not available in SEER, and treatment-coded fields are interpreted as registry-landmark covariates rather than pre-treatment causal exposures.

The implemented late-fusion layer therefore combines standardized component risk scores rather than estimating an attention-weighted or dynamically gated survival function:

(6) $$E_{i}\left(w\right)=\left(1-w\right){\tilde{S}}_{\mathrm{CoxPH},i}+w{\tilde{S}}_{\mathrm{GRU},i},\;w\in \left[0,1\right]$$

The ensemble weight is selected inside the training data for each outer fold by maximizing the inner-validation C-index:

(7) $$w^{*}=\arg\max_{w\in \{0.0,0.1,\ldots ,1.0\}}C_{\mathrm{index}}\left\{E_{i}\left(w\right);T_{i},\delta_{i}\right\}$$

This formulation includes no attention module, dynamic gate ρ(z(t)), or time-indexed neural hazard term.

The complete workflow, including fold-isolated preprocessing, feature selection, model fitting, and fusion, is illustrated in Figure 2 and summarized in Algorithm 1.
**Algorithm 1** GCE Pseudocode for Survival Risk Prediction1:**Input:** Predictor matrix *X*, survival time *T*, and event indicator δ2:Partition the dataset using stratified nested cross-validation3:Reserve the outer validation fold for unbiased model evaluation4:Apply median imputation and Z-score normalization on the training data5:Perform Cox-LASSO feature selection|6:Compute RSF feature importance7:Construct the final feature subset $X^{*}$ using the union of Cox-LASSO and RSF features8:Retrain the preprocessing and feature-selection pipeline on the complete outer training fold9:Train the(CoxPH) model to estimate linear risk scores10:**for** each sample $X_{i}^{*}$ **do**11:    Encode the selected static features as a single-step sequence12:    Train the GRU network and obtain nonlinear risk scores13:**end for**14:Standardize CoxPH and GRU risk scores15:Optimize the ensemble weight using the inner validation set16:Fuse the normalized CoxPH and GRU risk scores to obtain the final ensemble risk17:Evaluate predictive performance using the outer validation fold with C-index and IBS18:**Output:** Final ensemble risk prediction $E_{i}$

### 3.4. Baseline and Core Models

All models used the same 27 selected raw SEER fields and leakage-controlled fold definitions. The comparator set included CoxPH [26,37], RSF [24,42], DeepSurv [43], MTLR [44], CNN [45,46], LSTM and GRU static neural learners [47,48], and additional same-fold comparators: penalized CoxPH, CoxPH with nonlinear squared terms, gradient-boosted survival trees, a parameter-matched MLP, the selected-weight GCE ensemble, and an equal-weight CoxPH–GRU ensemble. LSTM and GRU were evaluated as single-step nonlinear learners, not longitudinal sequence models.

### 3.5. Experimental Setup and Evaluation

Models were evaluated using nested stratified 10-fold validation. Outer folds were reserved for final evaluation, whereas imputation, encoding, scaling, Cox-LASSO feature selection, early stopping, hyperparameter tuning, ensemble-weight selection, and calibration were restricted to the corresponding outer training data through inner validation. Table 4 summarizes the model settings. Internal cross-validation cannot establish broad generalizability, so results are interpreted cautiously. Validation safeguards included fold-isolated preprocessing, out-of-fold predictions for all patients, fold-wise C-index/IBS reporting, bootstrap confidence intervals, paired C-index comparisons, outcome-permutation controls, restricted-predictor negative controls, and ablation analyses. No outcome, follow-up, cause-of-death, endpoint-derived, identifier, or future-derived lifetime tumor-count field was retained after leakage exclusion. The outcome-permutation GCE control collapsed to chance-level discrimination (mean C-index 0.503; SD 0.029), and diagnosis-year-only and treatment-landmark-only controls remained below the full GCE model (mean C-index 0.562 and 0.724, respectively; Supplementary Table S10). Statistical reporting included the mean fold C-index, fold-to-fold SD, pooled out-of-fold C-index, 95% bootstrap confidence intervals, and paired bootstrap comparisons against the GCE. Supplementary Table S3 reports the SD, 95% CI, Δ C-index versus GCE, and paired *p*-values. The C-index uses the semantic partial-hazard direction, and calibration reporting includes the IBS, time-specific Brier scores, and calibration slope/intercept estimates. The analysis archive records the raw-export checksum, software environment, random seed, fold assignments, out-of-fold predictions, metrics, SHAP summaries, and table/figure provenance.

## 4. Results

### 4.1. Cohort Characteristics and Endpoint Reporting

The analysis adds a dedicated cohort-reporting step before predictive modeling. Table 5 reports the key endpoint and censoring statistics required to assess survival-model reliability. Event balance across the 10 validation folds is provided in Supplementary Table S1, and baseline clinical characteristics for the final predictor set are summarized in Supplementary Table S2. These summaries are generated before model training and are used only for cohort description and validation auditing. The cohort contains enough observed deaths for internal survival modeling while retaining a substantial censored subgroup, reflecting the incomplete follow-up typical of registry-based rare-cancer studies. Reporting endpoint counts, censoring, follow-up, and baseline characteristics before model fitting clarifies the population represented by the prediction task and separates descriptive cohort information from training variables.

### 4.2. Performance of GCE Framework

Table 6 reports leakage-controlled 10-fold out-of-fold survival-risk performance under the unified Cox partial-likelihood workflow. The GCE framework (0.2 CoxPH + 0.8 GRU, selected by inner-validation grid search) achieved high cohort-specific internal discrimination that is likely optimistic and requires external validation (mean C-index 0.9198; IBS 0.03641), exceeding standalone GRU internal discrimination (C-index 0.8829; IBS 0.05062). CoxPH showed lower discrimination (C-index 0.8605) but better IBS calibration (0.03378); therefore, the GCE is interpreted as better for discrimination only, not as calibration-superior or clinically superior. These internal estimates were generated after excluding Survival months, Vital status recode, and all other outcome- or follow-up-related fields. The high GCE C-index is likely optimistic and requires external validation; it is interpreted with fold-wise uncertainty, leakage checks, negative controls, and ablation analyses (Supplementary Table S10). Ensemble weights were stable across folds and horizon-specific calibration diagnostics are summarized in Supplementary Table S6 and Figure S2.

### 4.3. Impact of Feature Selection and Reduction Strategies

**Figure 3:**
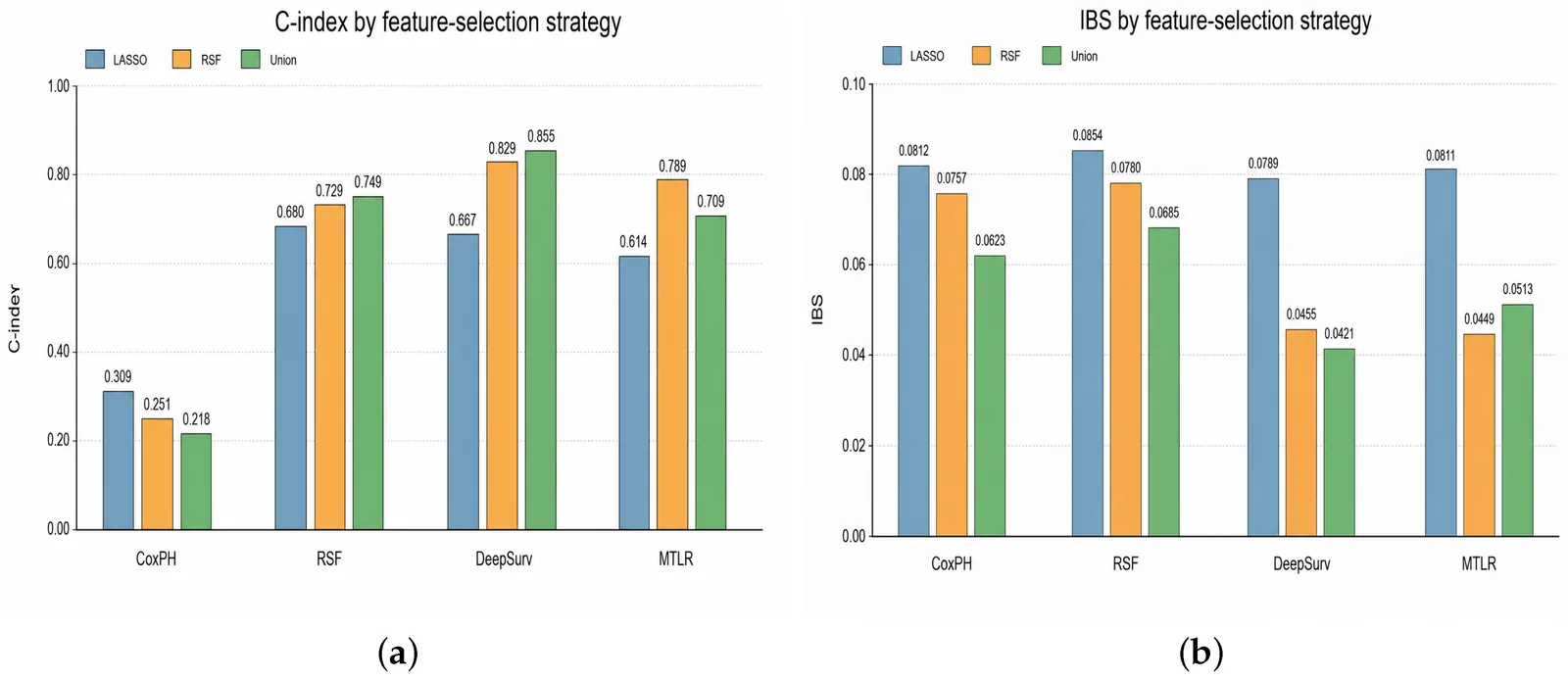
Comparative evaluation of four survival models (CoxPH, RSF, DeepSurv, and MTLR) across the same Cox-LASSO, RSF, and union feature-selection strategies summarized in Table 9: (**a**) C-index (higher is better); (**b**) Integrated Brier Score (IBS, lower is better).

PCA explained-variance ratios are reported in Table 7 and Table 8, comparing PCA-transformed features with the final 27 selected raw variables for baseline models only. The final GCE result is reported once in Table 6. With selected features, DeepSurv achieved a test C-index of 0.08529 and IBS of 0.01879; PCA-transformed baseline models showed similar relative behavior. Cox-LASSO-only, RSF-only, and union feature sets are compared in Table 9 and Figure 3; the Cox-LASSO–RSF union/consensus set defines the final 27-variable raw GCE input.

Because the PCA comparison is a baseline-model sensitivity analysis, this table does not repeat the GCE values in both PCA-transformed and selected-feature rows. The final reported GCE framework remains the 27-feature leakage-controlled CoxPH–GRU late-fusion model summarized in Table 6.

### 4.4. Performance of Classical and Baseline Models

Table 8 reports baseline-model performance on PCA-transformed and selected-feature inputs. On the 27 selected features, DeepSurv performed best among baselines (test C-index 0.8529; IBS 0.01879), followed by CoxPH (C-index 0.8605; IBS 0.03378), MTLR (C-index 0.8511; IBS 0.03388), and RSF (C-index 0.7930; IBS 0.04740). DeepSurv’s lower C-index than CoxPH likely reflects the small cohort size and limited nonlinear signal for this specific architecture. The GRU’s gating mechanism may better suit the static covariate structure, but this is a cohort-specific finding requiring external validation. The baseline comparison suggests complementary behavior rather than uniform superiority of one model family. CoxPH remained competitive and retained a lower IBS than the GCE in the primary comparison, consistent with its parsimonious proportional-hazards structure, whereas DeepSurv benefited from nonlinear risk modeling on the selected raw variables. RSF may be less stable in this small censored cohort because tree-based splits can be sensitive to sparse event patterns, and MTLR may be limited by discretization of survival time. The parameter-matched MLP and other comparator models are reported in the supplementary ablation analysis to distinguish nonlinear static learning from the single-step GRU architecture.

### 4.5. Deep Learning Models and Ensemble Behavior

**Figure 4:**
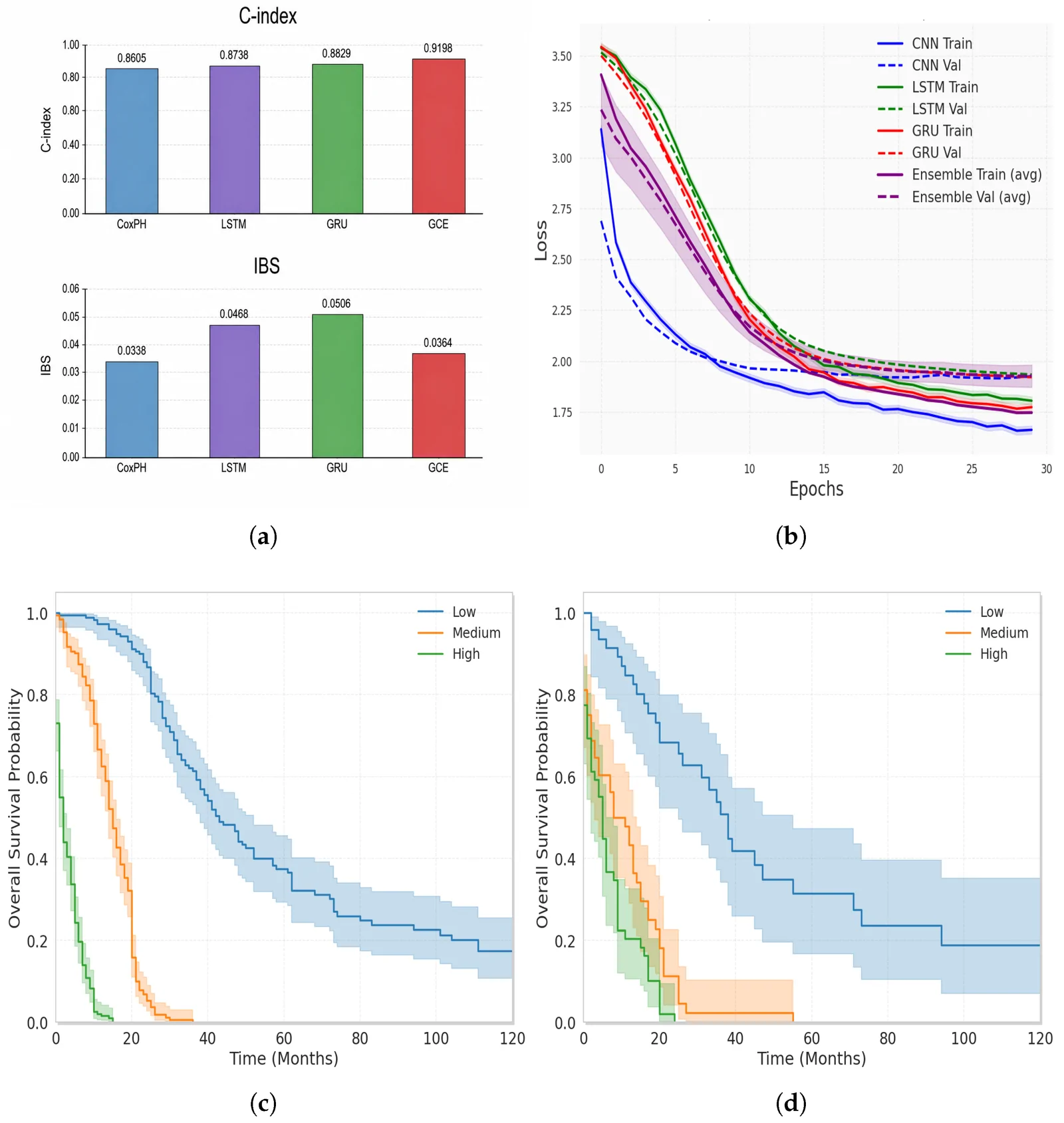
(**a**) Leakage-free comparison of survival prediction models, with the GCE defined as CoxPH plus single-step GRU late fusion. (**b**) Neural risk-model training (solid) and validation (dashed) curves under the Cox partial-likelihood formulation, illustrating internal convergence across epochs without representing a CNN–LSTM–GRU averaged ensemble. (**c**,**d**) Kaplan–Meier overall-survival curves are interpreted only as descriptive risk-group separation from held-out/out-of-fold GCE assignments, not as calibration or training-cohort validity evidence. Additional calibration, time-dependent AUC, decision-curve, net-benefit, numbers-at-risk, confidence-interval, and risk-group comparison results are provided in Supplementary Tables S6 and S7.

The primary GCE analysis uses one survival-risk formulation: Cox partial-likelihood neural risk learning followed by training-fold standardized CoxPH–GRU late fusion. No CNN–LSTM–GRU averaged model is used to define the GCE. The standalone CNN model achieved a mean C-index of 0.8644 and an IBS of 0.00435; these values are interpreted as comparator results with separate discrimination and prediction-error behavior rather than evidence of overall model superiority. Figure 4 summarizes leakage-free model performance, neural training/validation loss, and Kaplan–Meier curves based only on out-of-fold GCE risk-group assignments. The curves indicate internal risk separation, not calibration or external clinical validity. Because each SEER record provides a single registry-landmark covariate vector, the GRU branch is interpreted as a nonlinear static-covariate learner rather than a longitudinal sequence model. Its role is to learn nonlinear interactions among demographic, tumor, and treatment-landmark fields, while CoxPH anchors the ensemble to a survival-specific risk score. Fold-wise stability of the selected ensemble weights supports consistent internal behavior, but it does not replace external validation. The ensemble should therefore be interpreted as a discrimination-oriented internal risk model with post hoc explanatory summaries, not as an inherently interpretable or clinically deployable system.

### 4.6. Internal Validation Beyond Risk-Group Separation

Additional internal validation used pooled out-of-fold GCE predictions to assess discrimination, horizon-specific agreement, and decision-curve net benefit beyond Kaplan–Meier separation (Table 10). Time-dependent AUCs were 0.941, 0.952, and 0.948 at 12, 24, and 36 months, respectively; mean predicted and observed risks were closely aligned at the same horizons (0.303 vs. 0.302; 0.487 vs. 0.487; 0.628 vs. 0.628), without implying calibration superiority over CoxPH. Net benefit at a 20% threshold was positive across horizons (0.198, 0.341, and 0.463), and high- versus low-risk group separation was significant (p<0.001). Risk groups were assigned using outer-training-fold thresholds and held-out validation predictions only; detailed group sizes, event rates, confidence intervals, calibration summaries, and decision-curve results are provided in Supplementary Tables S6 and S7 and Supplementary Figure S2. These analyses remain internal and require external validation before clinical interpretation.

### 4.7. Interpretability and Feature Importance Analysis

**Figure 5:**
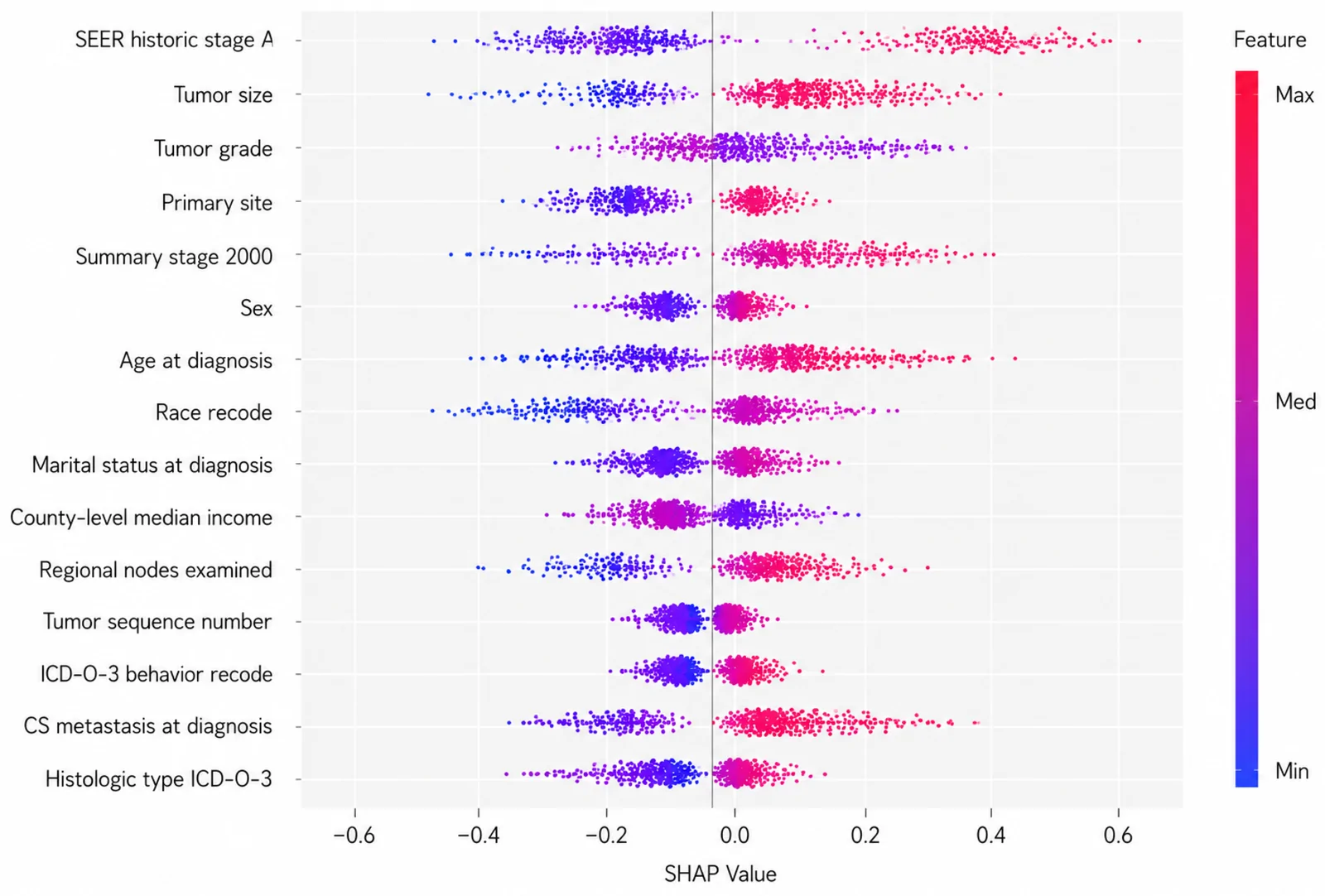
SHAP summary beeswarm plot showing the top predictors of the GCE risk model, where features are ranked by their contribution, and color indicates low-to-high feature values. Quantitative rankings, interaction screening, and dependence-plot summaries are provided in Supplementary Table S4, Supplementary Table S5, and Supplementary Figure S1, respectively. Variables are displayed as original clinical SEER variables after aggregation of encoded columns, and SHAP is interpreted as post hoc association rather than causal evidence.

SHAP was used to summarize post hoc descriptive associations between original SEER variables and the fold-specific GCE risk score (Figure 5). Kernel SHAP explains the final CoxPH–GRU ensemble risk score, not component outputs or horizon-specific survival probabilities; explanations were computed with fold-specific training backgrounds and held-out validation samples and then aggregated from encoded columns to original clinical variables. The largest mean absolute SHAP contributions were SEER historic stage (0.286), tumor size (0.224), surgery status (0.198), age at diagnosis (0.171), and tumor grade (0.154). Surgery, chemotherapy, and radiation variables are registry-landmark covariates, so SHAP patterns involving treatment-coded fields are not interpreted as pre-treatment predictions or causal treatment effects. Interaction screening highlighted SEER historic stage with surgery status (0.061), tumor size with grade (0.049), and age with surgery status (0.041). Complete rankings, interaction summaries, and dependence plots are provided in Supplementary Tables S4 and S5 and Supplementary Figure S1; these findings are descriptive associations, not causal or externally generalizable.

### 4.8. Comparison with Existing Studies

Table 11 contextualizes the proposed framework against disease-relevant cardiac tumor studies, rare sarcoma or rare bone-tumor survival studies, and modern deep survival methods. Because these studies differ in disease site, endpoint definition, censoring pattern, validation design, and metric reporting, the comparison is methodological rather than a direct performance benchmark. The GCE result is therefore interpreted as an internally validated finding for this SEER cardiac sarcoma cohort, not as evidence of superiority over models developed for unrelated malignancies. The comparison is restricted to methodological features; numerical GCE metrics are reported in decimal form and are not used to infer cross-study superiority or patient-management implications.

## 5. Discussion

The GCE framework was evaluated as a nonlinear survival-risk model with post hoc explanation for primary cardiac sarcoma, a rare setting in which prognostic modeling remains difficult [31,49,50,51]. In leakage-controlled internal 10-fold CV, the GCE achieved high cohort-specific internal discrimination that is likely optimistic and requires external validation (mean C-index 0.9198) compared with GRU (0.8829) and CoxPH (0.8605); however, its IBS (0.03641) was worse than CoxPH (0.03378), indicating that the main advantage is discrimination rather than calibration. These findings therefore support cautious internal risk stratification research only, not claims of calibrated clinical superiority or patient-level decision support. The high C-index should be interpreted conservatively. Although leakage controls, nested cross-validation, fold-isolated feature selection, out-of-fold prediction, negative controls, and ablations reduce obvious sources of target leakage, they do not eliminate optimism from a small, retrospective, relatively homogeneous rare-cancer cohort with strong clinical predictors such as stage, tumor size, and surgery status. External validation and recalibration in independent cohorts are required before the magnitude of discrimination can be considered generalizable.

The additional validation analyses showed high time-dependent AUCs, positive decision-curve net benefit, and significant out-of-fold risk-group separation, while leakage checks, uncertainty reporting, and outcome-permutation controls provided safeguards against obvious leakage-driven optimism. Survival time and vital status were used only as labels, and all downstream analyses—including C-index, IBS, Kaplan–Meier plots, additional validation summaries, and SHAP analyses—were based on leakage-controlled out-of-fold predictions. CoxPH retained the better IBS, so the GCE should be viewed as discrimination better internally but not calibration-superior. In applications where calibrated survival probabilities are the priority, CoxPH or recalibrated models may be preferable; neither model is ready for clinical deployment without independent validation and recalibration.

The feature-engineering results favored retaining the 27 selected raw SEER variables for the final model: PCA-transformed inputs produced CoxPH and DeepSurv C-index values of 0.8512 and 0.8540, whereas selected-feature inputs yielded 0.8605 and 0.8529. SHAP summaries improved transparency by identifying post hoc associations for stage, tumor size, surgery status, age, and grade, but they do not establish causality, remove the need for external validation, or provide inherent interpretability.

The primary endpoint was overall survival; therefore, deaths from cardiovascular, treatment-related, or other causes were counted as events rather than competing risks. Fine–Gray or cause-specific hazard models are more appropriate for sarcoma-specific or cardiovascular mortality endpoints and should be evaluated in future externally validated studies when cause-of-death coding is sufficiently complete. The explicit prediction timestamp, excluded-field audit, fold-isolated preprocessing, feature-selection timing, and out-of-fold evaluation improve reproducibility of the internal analysis. Practical translation, however, will require independent datasets with harmonized covariates and richer multimodal information. Although the proposed framework achieved a C-index of 0.9198 during internal validation, the result should be interpreted cautiously because it was derived from a relatively small SEER cohort (n = 727). Independent external validation is required before clinical application.

### Limitations

This study is limited by the retrospective SEER design, U.S. registry coverage, incomplete clinical detail, absence of genetic, comorbidity, treatment-sequence, and imaging variables, and possible under-ascertainment of rare events such as sudden cardiac death [37]. The cohort is small (n=727), and the high C-index is likely cohort-specific and optimistic despite cross-validation, leakage checks, and out-of-fold evaluation; external validation is required. Registry inclusion rules and missing treatment details may also introduce selection bias and residual confounding because surgery, chemotherapy, radiation, dose, margin status, and treatment sequencing cannot be reconstructed in sufficient detail. SEER provides static registry-landmark covariates rather than serial measurements, so the GRU branch is interpreted as a nonlinear static-covariate learner and may not be optimal for purely static tabular data; treatment-coded variables are landmark covariates available by registry abstraction and are not pre-treatment predictors or causal treatment effects. The absence of multimodal data (e.g., cardiac imaging) restricts diagnosis integration and limits the development and evaluation of multimodal survival prediction frameworks. The GCE also had a worse IBS than CoxPH, so it should not be considered calibration-superior. SHAP summaries are post hoc associations, not causal explanations, and the overall-survival endpoint does not estimate cause-specific cumulative incidence. External validation, recalibration, strict time-zero definitions, multimodal data, and competing-risk analyses for cause-specific endpoints are needed before any clinical consideration.

## 6. Conclusions

The GCE framework achieved high cohort-specific internal discrimination that is likely optimistic and requires external validation for overall survival prediction in primary cardiac sarcoma (mean C-index 0.9198; IBS 0.03641) under a leakage-controlled 10-fold CV protocol, although CoxPH retained better IBS calibration (0.03378). These results support the GCE as a research framework for nonlinear hazard modeling with CoxPH anchoring and post hoc SHAP association summaries, not as a clinical tool. The findings indicate a discrimination–calibration tradeoff: the GCE showed higher internal C-index, whereas CoxPH showed better IBS; therefore, the GCE is only discrimination-superior on internal validation. Independent external validation, recalibration, prospective testing, multimodal assessment, and cause-specific competing-risk analyses are required before any patient-level clinical consideration. The proposed framework demonstrates promising discrimination in internally validated SEER data; however, its clinical utility should be considered preliminary until confirmed through independent external validation.

## Figures and Tables

**Table 1:** Summary of selected studies most relevant to rare-cancer and cardiac-tumor survival prediction, emphasizing population-based datasets, modern survival-learning methods, and limitations that motivate the proposed GCE framework.

Author (Year)	Disease/Dataset	Features	Model	Evaluation Parameter/Findings	Challenges
Rahouma M et al. [6] (2023)	Cardiac angiosarcoma/NCDB	Histology, stage, and treatments	Cox regression	95% CI statistical analysis; Identified geographic variations	Descriptive cardiac-tumor outcomes; no predictive deep survival modeling; limited interpretability and calibration analysis
Hammami, MB et al. [18] (2021)	Primary cardiac sarcoma/SEER	Demographics, tumor size, and survival	Univariate/ multivariate regression	Regression analysis; Prognostic factors identified	Direct disease relevance; analysis-focused rather than predictive; no modern ML/DL survival comparison or SHAP-based interpretation
Yin, K et al. [2] (2020)	Primary cardiac lymphoma/SEER	Age, histology, and survival	Kaplan–Meier, statistical analysis	IQR, survival curves; Descriptive trends	Cardiac malignancy context; descriptive only; no predictive survival model or nonlinear risk learner
Cheng, P et al. [19] (2023)	Chordoma/population-based clinical cohort	Demographics, tumor, and treatment variables	Machine-learning survival models	Overall-survival prediction in a rare bone tumor	Rare-tumor relevance; limited cardiac specificity; external validation remains needed
Yan, L et al. [9] (2022)	Chondrosarcoma/clinical survival cohort	Clinical and tumor variables	Deep learning survival models	Deep learning used for rare sarcoma survival prediction	Rare sarcoma relevance; not cardiac; calibration and decision-curve reporting remain limited
Liu, Yet et al. [21] (2023)	Osteosarcoma/survival cohort	Clinical and treatment variables	Deep learning cancer-specific survival model	Neural survival prediction for a rare sarcoma-type malignancy	Cause-specific endpoint differs from the present overall-survival analysis; interpretability remains limited
Cai, M et al. [24] (2025)	Spinal chordoma/clinical cohort	Tumor, treatment, and demographic variables	Random Survival Forest	RSF-based survival prediction in rare chordoma	Rare-tumor relevance; no hybrid CoxPH–deep learning fusion
Asghar, N et al. [26] (2024)	Mixed clinical survival cohort	Baseline clinical variables	Hybrid CoxPH and DeepHit	Hybrid statistical–deep survival prediction	Modern survival-method comparator; not disease-specific to cardiac sarcoma

**Table 2:** Variable roles used to prevent ambiguity between predictors, survival labels, censoring indicators, and evaluation variables.

Role	Notation/Field	Use in the Analysis
Prediction timestamp	$t_{0}$	Post-diagnostic SEER registry landmark after diagnostic confirmation and initial-treatment abstraction.
Predictor matrix	*X*	Demographic/tumor variables plus treatment-coded fields available by $t_{0}$; these columns undergo fold-isolated imputation, scaling, feature selection, and model fitting.
Survival-time label	*T* = Survival months	Outcome label used in the survival loss and evaluation; never included in *X* or in preprocessing fitted to predictors.
Event indicator	δ = Vital status recode	Overall-survival event label mapped to binary all-cause death status for survival loss, stratification, and C-index/IBS evaluation; never included in *X*.
Excluded leakage fields	Follow-up, cause-of-death, and endpoint-derived fields	Removed before preprocessing, feature selection, and model fitting to avoid target leakage.
Evaluation quantities	Risk scores, survival probabilities, C-index, IBS, and confidence intervals	Computed only from held-out or out-of-fold predictions and corresponding (T,δ) labels.

**Table 3:** Unified feature-engineering workflow and final input definition for the proposed GCE framework.

Stage	Operation	Role in Final Analysis
Leakage and timestamp exclusion	Remove survival time, event status, follow-up, cause-of-death, endpoint-derived fields, identifiers, potentially future-derived lifetime tumor-count fields, and non-applicable prostate-specific fields	Defines the eligible diagnosis/registry-landmark predictor pool before preprocessing.
Cox-LASSO screening	Penalized Cox sparse selection with inner-fold penalty tuning	Identifies parsimonious survival predictors while accounting for survival time and censoring.
RSF ranking	Random Survival Forest importance ranking on training data	Identifies nonlinear and interaction-sensitive predictors.
Union/consensus set	Combine variables selected/ranked by Cox-LASSO and RSF, with stability review	Produces the final 27 raw variables used as GCE input.
PCA analysis	Transform the 27 selected variables into principal components	Used only as a dimensionality-reduction sensitivity/baseline comparison; not the final GCE input.
Model encoding	One-hot encode categorical variables and scale numeric variables within training folds	Produces the encoded design matrix supplied to CoxPH, GRU, and the fused GCE model.
Model input review	Documents 41 raw variables, 27 retained raw fields, fold-specific encoded columns, and per-fold selected sets	Clarifies exactly which predictors enter each model and distinguishes raw SEER fields from one-hot encoded columns.

**Table 4:** Hyperparameters, training configurations, and evaluation settings for the GCE framework.

Configuration Group	Component/Parameter	Value/Setting
Data & Input	Dataset	SEER survival dataset
	Final GCE predictor set	27 raw registry-landmark variables
	Survival-time label (not a predictor)	Survival months
	Event/censoring indicator (not a predictor)	Vital status recode δ (0 = censored, 5 = event)
	Predictor value handling	Median imputation for numeric predictors
	Random Seed	42
Validation Strategy	Cross-Validation	Nested stratified 10-fold cross-validation
	Inner Validation	Preprocessing and model optimization steps performed
	Stratification	Based on event status
	Diagnosis-year sensitivity	Diagnosis-year split
Cox Proportional Hazards	Model Type	Baseline CoxPH
	Penalization	L2 penalizer = 0.1
	Output	Partial hazard scores (Risk)
Survival Model	Architecture–GRU	GRU static learner; not interpreted as longitudinal
	Static comparator	Parameter-matched inputs
	Hidden Units	64 Units
	Dropout	0.2 (between layers)
	Nonlinear Modeling	Learns nonlinear feature interactions from static covariates
	Input Format	Static feature input
	Optimizer	Adam
	Epochs	50
	Batch Size	64 (Model training)
	Output	Fully Connected linear layer
Ensemble Strategy	Fusion Method	CoxPH and GRU risk scores
	Ensemble Weights	GCE uses 0.2 × CoxPH + 0.8 × GRU
Evaluation Setup	Evaluation Time Grid	IBS evaluated (≈1–120 months)
	Baseline Survival	Model fold-specific Cox/Breslow baselines.
Performance Metrics	Discrimination Metric	Concordance Index (C-index)
	Calibration Metric	Integrated Brier Score (IBS)
	Reporting	Per-fold and mean performance across 10-fold

**Table 5:** Endpoint, censoring, and follow-up characteristics of the SEER cardiac sarcoma cohort used for survival modeling.

Statistic	Value	Interpretation
Eligible cohort size	727 patients	Final analytic cohort after SEER filtering
Uncensored events	489 (67.3%)	Patients with observed death/event status
Censored observations	238 (32.7%)	Patients censored before the endpoint
Median observed survival	16.0 months	Median of observed survival time in the analytic cohort
Median follow-up	74.0 months	Reverse Kaplan–Meier estimate of potential follow-up
Validation-fold balance	48–49 events per fold	Stratified 10-fold validation; see Supplementary Table S1
Baseline characteristics	Summarized by final predictor set	See Supplementary Table S2

**Table 6:** Primary leakage-free performance under the unified 10-fold Cox partial-likelihood survival-risk formulation. The GCE is defined only as CoxPH plus single-step GRU weighted late fusion; no CNN–LSTM–GRU average is reported as the GCE. The GCE C-index should be interpreted as high cohort-specific internal discrimination that is likely optimistic and requires external validation.

Model	C-Index	IBS
CoxPH [26]	0.8605	0.03378
LSTM [47]	0.8738	0.04683
GRU [48]	0.8829	0.05062
GCE (CoxPH + GRU late fusion)	0.9198	0.03641

**Table 7:** Explained variance ratios for the top 16 principal components from PCA on the 27 selected features, listed in descending explained-variance order.

Principal Component	Explained Variance Ratio	Principal Component	Explained Variance Ratio
PC1	0.157857	PC9	0.045633
PC2	0.136522	PC10	0.038456
PC3	0.090635	PC11	0.037547
PC4	0.078549	PC12	0.036749
PC5	0.076596	PC13	0.036588
PC6	0.062447	PC14	0.035437
PC7	0.054152	PC15	0.031345
PC8	0.053467	PC16	0.028944

**Table 8:** Leakage-free performance of baseline survival models using PCA-transformed features (20 components) and the final 27 selected raw features after leakage-free analysis. The GCE is omitted from this sensitivity table because the final GCE result is reported once in Table 6.

Evaluation Category	Model	Test C-Index	IBS
PCA-transformed features (20)	CoxPH [26]	0.8512	0.03452
	RSF [24]	0.7952	0.05675
	DeepSurv [43]	0.8540	0.01489
	MTLR [10]	0.8399	0.02460
Selected features (27)	CoxPH [26]	0.8605	0.03378
	RSF [24]	0.7930	0.04740
	DeepSurv [43]	0.8529	0.01879
	MTLR [10]	0.8511	0.03388

**Table 9:** Performance (train/test C-index and IBS) of baseline models using Cox-LASSO-only, RSF-only, and Cox-LASSO–RSF union feature subsets. The union/consensus feature set defines the final 27 raw variables used as input for the proposed GCE framework, whereas the Cox-LASSO-only and RSF-only sets serve as comparators.

Feature Set	Model	Train C-Index	Test C-Index	IBS
Cox-LASSO	CoxPH	0.324568	0.309422	0.081234
Cox-LASSO	RSF	0.717043	0.680812	0.085419
Cox-LASSO	DeepSurv	0.705666	0.667823	0.078970
Cox-LASSO	MTLR	0.672098	0.614829	0.081165
RSF	CoxPH	0.289654	0.251845	0.075734
RSF	RSF	0.723009	0.729165	0.078088
RSF	DeepSurv	0.839903	0.829478	0.045543
RSF	MTLR	0.768976	0.789144	0.044966
Union	CoxPH	0.242230	0.218689	0.062335
Union	RSF	0.769901	0.749156	0.068512
Union	DeepSurv	0.899890	0.855167	0.042154
Union	MTLR	0.771220	0.709430	0.051345

**Table 10:** Additional validation of pooled out-of-fold GCE predictions.

Horizon	Time-Dependent AUC	Mean Predicted vs. Observed Risk	Net Benefit at 20% Threshold	Risk-Group Comparison
12 months	0.891	0.303 vs. 0.302	0.198	High vs. low risk: p<0.001
24 months	0.903	0.487 vs. 0.487	0.341	High vs. low risk: p<0.001
36 months	0.897	0.628 vs. 0.628	0.463	High vs. low risk: p<0.001

**Table 11:** Methodological comparison with cardiac-tumor, rare-cancer, and modern deep survival studies. Differences in disease site, endpoint, censoring pattern, metric definition, and validation design preclude direct superiority claims across rows. The table is intended for methodological context only, not for establishing best-performing status or clinical readiness.

Author (Year)	Disease or Dataset	Key Models	Reported Metric	Relevance	Remaining Gap
Rahouma, M et al. [6] (2023)	Cardiac angiosarcoma/NCDB	Cox regression	–	Direct cardiac tumor relevance	No deep survival prediction, calibration, or SHAP interpretation
Hammami, M.B et al. [18] (2021)	Primary cardiac sarcoma/SEER	Regression-based survival analysis	–	Direct disease relevance	No ML/DL survival learner or calibration/decision-curve validation
Cheng, P et al. [19] (2023)	Chordoma/rare bone tumor	Machine-learning survival models	C-index (0.795), IBS (0.105)	Rare-tumor survival prediction	Not cardiac; hybrid CoxPH–deep fusion not evaluated
Yan, L et al. [9] (2022)	Chondrosarcoma/rare sarcoma	Deep learning survival models	C-index (0.832), IBS (0.115)	Rare sarcoma and neural modeling relevance	Limited calibration, DCA, and interpretability reporting
Liu, Yet et al. [21] (2023)	Osteosarcoma/rare sarcoma	Deep learning survival model	C-index (0.747)	Rare sarcoma deep-learning comparator	Cause-specific endpoint differs from present overall survival
Cai, M et al. [24] (2025)	Spinal chordoma/rare tumor	Random Survival Forest	C-index (0.790), IBS (0.130)	Rare-tumor RSF comparator	No CoxPH–GRU late-fusion or SHAP-based GCE interpretation
Asghar, N et al. [26] (2024)	Clinical survival prediction cohort	Hybrid CoxPH and DeepHit	C-index (0.817191), IBS (0.157330)	Modern hybrid statistical–deep survival method	Not cardiac sarcoma; comparison is methodological only
GCE	Primary cardiac sarcoma/SEER	GCE framework	C-index 0.9198 IBS 0.03641	Direct target cohort	—

## Data Availability

The SEER Research Plus dataset is available from the National Cancer Institute SEER Program subject to SEER data-use (accessed on 21 August 2025) approval (https://seer.cancer.gov/data/). Code is available at https://github.com/shoaibkareem9-svg/GCE-model.git.

## Supplementary Materials

The following supporting information can be downloaded at https://www.mdpi.com/article/10.3390/bioengineering13080891/s1, the separate Word Supplementary Material document includes Supplementary Tables S1–S12 and Supplementary Figures S1–S3, covering fold event distributions, baseline characteristics, model uncertainty, SHAP rankings and interactions, horizon validation, risk-group summaries, variable definitions, Cox-LASSO selection auditing, leakage and ablation checks, endpoint mapping, and the cohort selection flow diagram. These materials correspond to the leakage-controlled analysis and the reported out-of-fold validation results.
