# Supplementary material for: GCE: A Framework for Interpretable Nonlinear Hazard Modeling in Cardiac Sarcoma Survival Using SEER Data
Source: Bioengineering (Basel). 2026 Aug 1;13(8):891. doi: 10.3390/bioengineering13080891 (PMC13509387; doi:10.3390/bioengineering13080891)
Supplement: Supplementary file 1 [file bioengineering-13-00891-s001.zip › bioengineering-4443125-supplementary.pdf]

Supplementary File

GCE: A CoxPH–GRU Framework for Nonlinear Hazard Modeling in Cardiac Sarcoma Survival Using SEER Data

Muhammad Shoaib Kareem[1] , Madiha Amjad[1,2], , Saba Aslam[3] , Abdur Rasool[4]∗ , Mutiullah Jamil[1] , Hazrat Ali[5]∗

[1]Department of Information Technology, Khawaja Fareed University of Engineering and Technology, RahimYar Khan, 64200, Pakistan; shoaibkareem8@gmail.com ; inft201700004@kfueit.edu.pk (S.K.)

[2]Department of Computer Science, COMSATS University Islamabad, Lahore Campus,54792, Pakistan;

[3]Shenzhen Institutes of Advanced Technology, Chinese Academy of Sciences, Shenzhen, 518055, China

[4] Department of Information and Computer Sciences, University of Hawaii at Manoa, Honolulu, HI 96822, USA

[5]Division of Computing Science and Mathematics, University of Stirling, Stirling, FK9 4LA, United Kingdom

* Correspondence: Email: abdur@hawaii.edu (Abdur Rasool), hazrat.ali@live.com (Hazrat Ali)

Table of Contents

S1.Distribution of Validation Patients, Observed Death Events, and Censoring Across Stratified Cross-Validation Folds of the SEER Cardiac Sarcoma Cohort ……………………………………………3

S2.Baseline Demographic, Clinical, Treatment, and Survival Characteristics of the SEER Cardiac Sarcoma Cohort ………………………………………………………………………… 4

S3. Fold-wise Performance, Uncertainty, and Statistical Comparison of Survival Models Acros 10-Fold Cross-Validation……………………………………………………..……………………..5

S4. Global SHAP-Based Feature Importance of Original Clinical Variables in the Final GCE Framework……………………………………………………………………………………6

S5. SHAP Dependency and Interaction Analysis of the Highest-Ranked Original Clinical Variables in the GCE Framework……………………………………………………………………………..7

S6. Time-Dependent Validation Metrics for the GCE Survival Prediction Framework… …………8

S7. Validation-Based Risk Stratification Performance of the GCE Framework Across Low-, Intermediate-, and High-Risk Groups…………………………………………………. ………..9

S8. Raw SEER Variable Screening and Leakage-Free Predictor Selection for the Final GCE Framework………………………………………………………………………………. ………..11

S9. Cox-LASSO fold-wise feature-selection and encoded-input audit……………………. ………...15

S10. Quality-Control Analyses for Verification of the Leakage-Free GCE Survival Modeling Workflow………………………………………………………………………. …………… ...20

S11. Reproducible Cohort Selection, Endpoint Definition, and Censoring Strategy for the SEER Cardiac Sarcoma Cohort………………………………………………………………… ………………24

Figure S1. SHAP dependence patterns for the final GCE risk score…………………………………...26

Figure S2. Calibration and decision-curve assessment of GCE………………………………………...27

Figure S3. Cohort selection flow diagram……………………………………………………….…..….28

Table S12. Cross-reference between supplementary materials and their corresponding sections in the main manuscript……………………… …………………………………………………..……..29

S1. Distribution of Validation Patients, Observed Death Events, and Censoring Across Stratified Cross-Validation Folds of the SEER Cardiac Sarcoma Cohort

This table reports the number of validation patients, observed death events, censored observations, and event percentages in each fold of the internally validated SEER cardiac sarcoma cohort (n = 727). The split was stratified by event status so that discrimination, calibration, and uncertainty estimates for the proposed GCE research framework were evaluated under comparable censoring conditions across folds.

For each validation fold, Event % was calculated as (number of observed events / validation patients) x 100, and Censored was calculated as validation patients - observed events. These quantities describe the effective survival information available in each held-out fold. Comparable event and censoring proportions are important because the C-index and time-dependent prediction-error estimates depend on the ordering and observability of survival times; this table therefore supports cautious internal validation of the GCE framework.

Supplementary Table S1. Event and censoring distribution across 10 out-of-fold validation splits.

Fold	Validation patients	Events	Censored	Event %
1	73	49	24	67.1
2	73	49	24	67.1
3	73	49	24	67.1
4	73	49	24	67.1
5	73	49	24	67.1
6	73	49	24	67.1
7	73	49	24	67.1
8	72	49	23	68.1
9	72	49	23	68.1
10	72	48	24	66.7
Total	727	489	238	67.3

.

S2. Baseline Demographic, Clinical, Treatment, and Survival Characteristics of the SEER Cardiac Sarcoma Cohort

This table summarizes demographic, tumor, treatment, and survival-endpoint characteristics before model development. Counts and percentages are calculated using the full analytic cohort denominator unless otherwise stated; unknown or missing clinical categories are retained as explicit categories to preserve cohort size and avoid exclusion-based selection bias.

Continuous variables are summarized using median and interquartile range (IQR), where the IQR equals the 75th percentile minus the 25th percentile. Categorical variables are summarized as count (%), where the percentage was calculated as (category count / 727) × 100.These baseline descriptors define the diagnosis-time clinical information available to all models and confirm that the proposed GRU-CoxPH framework was evaluated on routinely available SEER variables rather than outcome-derived fields.

Supplementary Table S2. Baseline clinical characteristics of the analytic SEER cardiac sarcoma cohort.

Characteristic	Summary
Age at diagnosis	Median 62 years (IQR 49-73)
Age group	<=65 years: 397 (54.6%); >65 years: 330 (45.4%)
Sex	Male: 379 (52.1%); Female: 348 (47.9%)
Race/ethnicity	White: 567 (78.0%); Black: 82 (11.3%); Other/unknown: 78 (10.7%)
Diagnosis period	2000-2009: 255 (35.1%); 2010-2016: 240 (33.0%); 2017-2022: 232 (31.9%)
Histologic subtype	Angiosarcoma: 278 (38.2%); Sarcoma NOS: 183 (25.2%); Leiomyosarcoma: 89 (12.2%); Rhabdomyosarcoma: 41 (5.6%); Other: 136 (18.7%)
SEER historic stage	Localized: 169 (23.2%); Regional: 213 (29.3%); Distant: 276 (38.0%); Unknown: 69 (9.5%)
Tumor grade	I-II: 93 (12.8%); III-IV: 347 (47.7%); Unknown: 287 (39.5%)
Tumor size	<=4 cm: 181 (24.9%); >4 cm: 386 (53.1%); Unknown: 160 (22.0%)
Surgery	Yes: 403 (55.4%); No/unknown: 324 (44.6%)
Chemotherapy	Yes: 318 (43.7%); No/unknown: 409 (56.3%)
Radiotherapy	Yes: 162 (22.3%); No/unknown: 565 (77.7%)
Household income quartile	Q1: 167 (23.0%); Q2: 178 (24.5%); Q3: 187 (25.7%); Q4: 195 (26.8%)

S3. Fold-wise Performance, Uncertainty, and Statistical Comparison of Survival Models Acros 10-Fold Cross-Validation

This table reports fold-wise uncertainty for each survival model using leakage-free 10-fold cross-validation outputs. Mean C-index, C-index standard deviation, bootstrap 95% confidence intervals, integrated Brier score (IBS), C-index difference versus GCE, and paired bootstrap p-values quantify internal discrimination, prediction-error calibration, and comparative uncertainty.

The mean C-index is the average fold-wise concordance estimate; SD is the fold-to-fold standard deviation of C-index, not IBS. The 95% CI summarizes bootstrap uncertainty in the out-of-fold C-index, Delta C-index versus GCE equals C-index_model - C-index_GCE, and the paired p-value compares paired discrimination differences against GCE. IBS represents the integrated time-dependent Brier score, with lower values indicating lower prediction error over follow-up. The table supports cautious interpretation: GCE has the highest internal discrimination, whereas CoxPH has the better IBS.

Supplementary Table S3. Model performance uncertainty and paired comparisons using leakage-free out-of-fold predictions.

**Model**	**Mean C-index ↑**	**SD**	**95% CI**	**IBS ↓**	**Delta C-index vs. GCE**	**p vs. GCE**
GCE	**0.9198**	0.0311	0.901 –0.938	0.03641	0.0000	N/A(GCE comparator)
GRU	0.8829	0.0367	0.861 – 0.905	0.05062	-0.0369	0.008
LSTM	0.8738	0.0384	0.850 – 0.897	0.04683	-0.0460	0.005
DeepSurv	0.8529	0.0411	0.827 – 0.878	0.01879	-0.0669	<0.001
CoxPH	0.8605	0.0427	0.834 – 0.886	0.03378	-0.0593	0.002
MTLR	0.8511	0.0455	0.823 – 0.878	0.03388	-0.0687	<0.001
RSF	0.7930	0.0511	0.762 – 0.823	0.04740	-0.1268	<0.001

S4. Global SHAP-Based Feature Importance of Original Clinical Variables in the Final GCE Framework

This table ranks the aggregated original clinical variables by mean absolute SHAP contribution to the final out-of-fold GCE risk score. The analysis provides post-hoc descriptive association summaries after leakage-related variables were excluded and feature selection was restricted to training folds.

For each variable, SHAP values decompose an individual prediction into feature-level contributions to the GCE risk score. Mean absolute SHAP values average contribution magnitudes across patients, while directionality summaries describe whether higher feature values tend to increase or decrease predicted risk. These summaries are post-hoc associations, not causal effects or proof of inherent clinical interpretability.

Supplementary Table S4. Final-ensemble SHAP ranking for the corrected GCE risk score.

Rank	Clinical variable	Mean |SHAP|	Dominant direction of association	Clinical interpretation
1	SEER historic stage	0.286	Distant/regional stage increased predicted risk	Extent of disease was the strongest contributor, consistent with advanced cardiac sarcoma carrying poorer survival.
2	Tumor size	0.224	Larger tumors increased predicted risk	Greater local tumor burden likely reflects biologically aggressive or less resectable disease.
3	Surgery status	0.198	No surgery/unknown increased predicted risk	This is interpreted as an association with treatment eligibility and disease severity, not as a causal treatment effect.
4	Age at diagnosis	0.171	Older age increased predicted risk	Older patients may have reduced physiologic reserve and fewer tolerable treatment options.
5	Tumor grade	0.154	High grade increased predicted risk	Poor differentiation is clinically consistent with aggressive tumor biology.
6	Histologic subtype	0.132	Angiosarcoma/Sarcoma NOS showed higher risk patterns	Subtype-specific risk is plausible because cardiac sarcomas differ in aggressiveness and treatment response.
7	Chemotherapy status	0.106	Mixed, treatment-pattern dependent	The association likely reflects treatment selection and disease burden rather than causal benefit or harm.
8	Year of diagnosis	0.083	Later years showed modest risk attenuation	This may reflect secular changes in diagnosis, coding, or management and requires cautious interpretation.

S5. SHAP Dependency and Interaction Analysis of the Highest-Ranked Original Clinical Variables in the GCE Framework

This table summarizes descriptive SHAP dependency patterns among the highest-ranked original clinical variables. Interaction-screen values describe co-varying contributions to the GCE risk score and are intended for post-hoc model interpretation rather than causal treatment-effect inference.

The dependency screen was generated from SHAP dependence patterns after aggregation to clinically interpretable original variables. The interaction-strength summaries describe whether the SHAP contribution of one feature varies across levels of another feature; they do not estimate causal interactions or treatment effects. This table helps evaluate whether the GRU-CoxPH ensemble captures plausible nonlinear associations while preserving cautious, non-causal interpretation.

Supplementary Table S5. SHAP dependency screen for clinically interpretable feature interactions.

**Rank**	**Feature pair**	**Interaction screen strength**	**Observed dependency pattern**	**Interpretation**
1	SEER historic stage x Surgery status	0.061	Distant disease combined with no surgery/unknown produced the largest positive SHAP shifts.	Suggests that disease extent and operability jointly influence predicted risk.
2	Tumor size x Tumor grade	0.049	Large tumors with high grade showed amplified risk contributions.	Consistent with tumor burden and aggressive biology acting together.
3	Age at diagnosis x Surgery status	0.041	Older nonsurgical patients showed higher risk contributions.	May reflect frailty, comorbidity, or treatment-selection effects not directly captured in SEER.
4	Histologic subtype x SEER historic stage	0.037	High-risk histologies had stronger positive contributions when regional/distant disease was present.	Indicates that subtype effects should be interpreted in the context of disease extent.
5	Diagnosis period x Treatment variables	0.029	Later diagnosis years modestly modified treatment-associated SHAP patterns.	May reflect temporal changes in coding or care pathways rather than temporal patient sequences.

S6.Time-Dependent Validation Metrics for the GCE Survival Prediction Framework

This table reports time-dependent discrimination, prediction error, calibration, and decision-curve diagnostics at 12, 24, and 36 months using pooled out-of-fold GCE predictions. These are internal validation diagnostics; patients censored before a horizon were handled according to the time-dependent survival metric definition used for that endpoint.

Time-dependent AUC summarizes horizon-specific internal discrimination, while the Brier score at time t evaluates prediction error with censoring adjustment as implemented in the survival-evaluation procedure. Calibration intercept and slope assess agreement between predicted and observed survival risk, and decision-curve net benefit is interpreted descriptively under internal validation. Event probability is expected to increase from 12 to 36 months because cumulative incidence rises as follow-up time lengthens and more events become observable; these diagnostics do not establish calibration superiority over CoxPH or external validity.

Supplementary Table S6. Horizon-specific validation diagnostics for the GCE framework.

Horizon	Comparable patients	Events by horizon	Time-dependent AUC	Mean predicted risk	Observed event rate	Net benefit at 20% threshold	Time-specific Brier score	Calibration intercept	Calibration slope
12 months	676	204	0.941	0.303	0.302	0.246	0.071	0.02	0.91
24 months	641	312	0.952	0.487	0.487	0.414	0.083	-0.03	0.86
36 months	603	379	0.948	0.628	0.628	0.557	0.096	-0.06	0.82

S7. Validation-Based Risk Stratification Performance of the GCE Framework Across Low-, Intermediate-, and High-Risk Groups

This table reports low-, intermediate-, and high-risk strata reconstructed only from validation-fold GCE predictions. Risk thresholds were derived within each outer training fold, applied unchanged to the corresponding validation fold, and pooled after assignment to assess internal risk stratification without using validation outcomes to define groups.

Risk groups were assigned from validation-fold predictions only, and event rates were calculated as events / patients in each group x 100. Confidence intervals quantify uncertainty in the observed group-specific event proportions, and the log-rank p-value tests equality of survival curves across risk strata. Monotonic separation across low-, intermediate-, and high-risk groups supports internal discrimination of the GCE risk score without establishing calibration superiority or clinical deployability.

Supplementary Table S7. Out-of-fold risk-group assignment, event-rate uncertainty, and survival separation.

Panel	Risk group / time point	Patients or n at risk	12-month event rate (95% CI)	24-month event rate (95% CI)	36-month event rate (95% CI)	Statistical comparison / note
A. Fold-derived OOF risk groups	Low	238	8.8% (5.3-12.4)	18.9% (14.0-23.9)	31.8% (25.9-37.7)	Baseline risk group for log-rank comparison
A. Fold-derived OOF risk groups	Intermediate	244	27.0% (21.4-32.5)	47.1% (40.8-53.4)	63.1% (57.0-69.1)	High vs. low: log-rank $p < 0.001$
A. Fold-derived OOF risk groups	High	245	55.5% (49.3-61.8)	80.8% (75.8-85.7)	92.2% (88.8-95.5)	High vs. intermediate: log-rank $p < 0.001$
B. Numbers at risk	0 months	Low: 238; Intermediate: 244; High: 245	N/A	N/A	N/A	Validation-fold assignments pooled after threshold application
B. Numbers at risk	12 months	Low: 214; Intermediate: 190; High: 118	N/A	N/A	N/A	Counts computed from held-out/out-of-fold assignments
B. Numbers at risk	24 months	Low: 186; Intermediate: 132; High: 67	N/A	N/A	N/A	Counts computed from held-out/out-of-fold assignments

B. Numbers at risk	36 months	Low: 154; Intermediate: 86; High: 31	N/A	N/A	N/A	Counts computed from held-out/out-of-fold assignments
B. Numbers at risk	60 months	Low: 107; Intermediate: 43; High: 9	N/A	N/A	N/A	Counts computed from held-out/out-of-fold assignments
B. Numbers at risk	120 months	Low: 38; Intermediate: 8; High: 1	N/A	N/A	N/A	Counts computed from held-out/out-of-fold assignments
C. Threshold protocol	Fold-specific thresholds	10 outer folds	N/A	N/A	N/A	Each validation fold used only thresholds estimated from its corresponding outer training fold

S8. Raw SEER Variable Screening and Leakage-Free Predictor Selection for the Final GCE Framework

This table lists all raw SEER variables reviewed for the revised leakage-free workflow, including retained diagnosis-time predictors and excluded outcome, follow-up, identifier, future-derived, non-applicable, or high-missingness variables. It documents how the final predictor matrix was defined before imputation, encoding, scaling, and training-fold-only feature selection.

Each row documents whether a raw SEER field was retained as a diagnosis-time predictor or excluded because it represented an outcome, follow-up variable, identifier, future-derived field, non-applicable item, or high-missingness variable. This table is central to leakage prevention: outcome variables such as survival time, vital status, cause of death, and follow-up-derived fields are excluded before preprocessing. The retained variables define the candidate feature space evaluated by Cox-LASSO and the downstream GRU-CoxPH ensemble.

Supplementary Table S8. SEER data dictionary, exclusion rationale, and preprocessing plan.

No.	Raw SEER variable	Variable group	Model status	Encoding plan	Reason / availability note
1	SEER historic stage A	Tumor extent/stage	Retained final raw field	Categorical one-hot	Selected prognostic stage descriptor available by registry landmark.
2	Tumor size	Tumor burden	Retained final raw field	Numeric scaled	Tumor burden predictor available at diagnosis/registry abstraction.
3	Surgery status	Initial treatment landmark	Retained final raw field	Categorical one-hot	Treatment-coded landmark predictor; not interpreted as pre-treatment input.
4	Age at diagnosis	Demographic	Retained final raw field	Numeric scaled	Core demographic survival predictor.
5	Histologic type ICD-O-3	Tumor pathology	Retained final raw field	Categorical one-hot	Histopathologic subtype information.
6	Tumor grade	Tumor pathology	Retained final raw field	Categorical one-hot	Tumor differentiation/severity descriptor.
7	Chemotherapy recode	Initial treatment landmark	Retained final raw field	Categorical one-hot	Initial-treatment landmark field.
8	Primary site	Tumor site	Retained final raw field	Categorical one-hot	Anatomic primary site descriptor.
9	Summary stage 2000	Tumor extent/stage	Retained final raw	Categorical one-hot	SEER stage summary available by registry

No.	Raw SEER variable	Variable group	Model status	Encoding plan	Reason / availability note
			field		abstraction.
10	Surgery of primary site	Initial treatment landmark	Retained final raw field	Categorical one-hot	Surgical procedure code available by treatment abstraction.
11	Radiation recode	Initial treatment landmark	Retained final raw field	Categorical one-hot	Initial-treatment landmark field.
12	Sex	Demographic	Retained final raw field	Categorical one-hot	Core demographic variable.
13	Diagnostic confirmation	Registry/pathology	Retained final raw field	Categorical one-hot	Pathologic/diagnostic evidence descriptor.
14	CS extension	Tumor extent/stage	Retained final raw field	Categorical one-hot	Collaborative-stage local extension descriptor.
15	CS metastasis at diagnosis	Tumor extent/stage	Retained final raw field	Categorical one-hot	Metastatic status at diagnosis.
16	Year of diagnosis	Administrative calendar encoding	Retained final raw field	Numeric scaled	Calendar-time encoding; not individual follow-up duration.
17	Race recode	Demographic	Retained final raw field	Categorical one-hot	Population demographic predictor.
18	Reason no cancer-directed surgery	Initial treatment landmark	Retained final raw field	Categorical one-hot	Treatment-abstraction field interpreted only at registry landmark.
19	Marital status at diagnosis	Demographic/social	Retained final raw field	Categorical one-hot	Social/demographic descriptor commonly used in SEER survival prediction.
20	Radiation sequence with surgery	Initial treatment landmark	Retained final raw field	Categorical one-hot	Treatment sequencing field available by registry abstraction.
21	CS lymph nodes	Tumor extent/stage	Retained final raw field	Categorical one-hot	Nodal involvement descriptor.
22	County-level median income	Area-level socioeconomic	Retained final raw field	Numeric or ordinal scaled	Area-level socioeconomic covariate.
23	Laterality	Tumor site	Retained final raw field	Categorical one-hot	Tumor laterality descriptor where applicable.

No.	Raw SEER variable	Variable group	Model status	Encoding plan	Reason / availability note
24	Regional nodes examined	Registry/pathology	Retained final raw field	Numeric scaled	Pathology/staging intensity descriptor.
25	Tumor sequence number	Registry landmark/history	Retained final raw field	Ordinal/categorical encoded	Prior tumor sequence/history descriptor available by registry abstraction.
26	ICD-O-3 behavior recode	Tumor pathology	Retained final raw field	Categorical one-hot	Behavior code restricted to malignant analytic cohort but retained for audit consistency.
27	Regional nodes positive	Registry/pathology	Retained final raw field	Numeric scaled	Nodal positivity descriptor.
28	Patient ID	Identifier	Excluded before preprocessing	Not encoded	Registry identifier; not a predictor.
29	Survival months	Survival label	Excluded before preprocessing	Not encoded	Used only as survival time T.
30	Survival months recode	Outcome/follow-up derived	Excluded before preprocessing	Not encoded	Duplicate or derived survival-time field.
31	Vital status recode	Event/censoring label	Excluded before preprocessing	Not encoded	Used only to construct event indicator delta.
32	Vital status recode (study cutoff used)	Event/censoring label	Excluded before preprocessing	Not encoded	Alternative event/censoring label field.
33	Year of follow-up recode	Follow-up field	Excluded before preprocessing	Not encoded	Follow-up information unavailable at prediction timestamp.
34	SEER cause-specific death classification	Cause-of-death endpoint	Excluded before preprocessing	Not encoded	Endpoint/cause-of-death audit field only.
35	COD to site recode	Cause-of-death endpoint	Excluded before	Not encoded	Endpoint/cause-of-death audit field only.

No.	Raw SEER variable	Variable group	Model status	Encoding plan	Reason / availability note
			preprocessing		
36	Total number of in situ/malignant tumors for patient	Potentially future-derived	Excluded before preprocessing	Not encoded	Lifetime tumor-count field may contain information beyond the index landmark.
37	Prostate-specific age-standard field	Non-applicable derived field	Excluded before preprocessing	Not encoded	Cancer-site-specific/implausible field for cardiac sarcoma; removed after audit.
38	Detailed radiation dose/sequence field	High-missingness treatment detail	Excluded before preprocessing	Not encoded	Detailed treatment field excluded a priori because missingness exceeded 20%.
39	Detailed chemotherapy sequence field	High-missingness treatment detail	Excluded before preprocessing	Not encoded	Detailed treatment field excluded a priori because missingness exceeded 20%.
40	Comorbidity index field	Unavailable/incomplete SEER-Medicare detail	Excluded before preprocessing	Not encoded	Incomplete or unavailable for the analytic SEER cohort.
41	Cancer-specific survival indicator	Endpoint-derived field	Excluded before preprocessing	Not encoded	Endpoint-derived variable not used for overall-survival prediction.

S9. Cox-LASSO fold-wise feature-selection and encoded-input audit.

This consolidated audit documents the Cox-LASSO feature-selection process and the encoded model-input definition used by the revised GCE framework. The explicitly reported performance values are reproduced exactly from the manuscript, while fold-wise selection summaries document stability of the training-fold-only feature-selection procedure.

The Cox-LASSO selection step minimizes the penalized partial-likelihood objective within each training fold, where controls sparsity and the sum of absolute coefficients. Reported fold-wise penalties and selected-feature counts document that feature selection was performed independently within training data before validation prediction. This audit supports reproducibility and verifies that the GCE framework did not select predictors using held-out outcomes.

Table S9A. Fold-wise Cox-LASSO penalty and selected-feature summary.

Outer fold	Selected Cox-LASSO penalty (lambda)	Inner-validation C-index	Selected raw features	Selected encoded columns	Outer fold use
1	0.010	0.711	20	37	Evaluation only after inner selection and refit
2	0.030	0.698	21	37	Evaluation only after inner selection and refit
3	0.010	0.723	20	38	Evaluation only after inner selection and refit
4	0.003	0.736	21	38	Evaluation only after inner selection and refit
5	0.010	0.704	20	36	Evaluation only after inner selection and refit
6	0.030	0.719	20	38	Evaluation only after inner selection and refit
7	0.010	0.727	20	37	Evaluation only after inner selection and refit
8	0.010	0.708	21	37	Evaluation

Outer fold	Selected Cox-LASSO penalty (lambda)	Inner-validation C-index	Selected raw features	Selected encoded columns	Outer fold use
					only after inner selection and refit
9	0.003	0.732	21	39	Evaluation only after inner selection and refit
10	0.030	0.716	21	38	Evaluation only after inner selection and refit

Raw-feature selection frequency was calculated as the number of outer folds in which a feature was selected divided by 10, expressed as a percentage when reported. Features selected in most folds represent stable registry-landmark predictors under the Cox-LASSO screening step, whereas lower-frequency variables indicate fold-dependent evidence. This table clarifies which variables most consistently entered the nonlinear survival ensemble.

Table S9B. Raw-feature selection frequency across the 10 outer folds.

Rank	Raw SEER feature	Variable category	Folds selected	Selection frequency	Selected folds
1	SEER historic stage A	Tumor extent/stage	10/10	1.00	1, 2, 3, 4, 5, 6, 7, 8, 9, 10
2	Tumor size	Tumor burden	10/10	1.00	1, 2, 3, 4, 5, 6, 7, 8, 9, 10
3	Surgery status	Initial treatment landmark	10/10	1.00	1, 2, 3, 4, 5, 6, 7, 8, 9, 10
4	Age at diagnosis	Demographic	10/10	1.00	1, 2, 3, 4, 5, 6, 7, 8, 9, 10
5	Histologic type ICD-O-3	Tumor pathology	10/10	1.00	1, 2, 3, 4, 5, 6, 7, 8, 9, 10
6	Tumor grade	Tumor pathology	9/10	0.90	1, 2, 3, 4, 5, 7, 8, 9, 10
7	Chemotherapy recode	Initial treatment landmark	9/10	0.90	1, 2, 3, 4, 5, 6, 8, 9, 10
8	Primary site	Tumor site	9/10	0.90	1, 2, 3, 4, 5, 6, 7, 9, 10
9	Summary stage 2000	Tumor extent/stage	9/10	0.90	1, 2, 3, 4, 5, 6, 7, 8, 10
10	Surgery of primary site	Initial treatment landmark	9/10	0.90	1, 2, 3, 4, 5, 6, 7, 8, 9
11	Radiation recode	Initial treatment landmark	8/10	0.80	2, 4, 5, 6, 7, 8, 9, 10

Rank	Raw SEER feature	Variable category	Folds selected	Selection frequency	Selected folds
12	Sex	Demographic	8/10	0.80	1, 3, 5, 6, 7, 8, 9, 10
13	Diagnostic confirmation	Registry/pathology	8/10	0.80	1, 2, 4, 6, 7, 8, 9, 10
14	CS extension	Tumor extent/stage	8/10	0.80	1, 2, 3, 5, 7, 8, 9, 10
15	CS metastasis at diagnosis	Tumor extent/stage	8/10	0.80	1, 2, 3, 4, 6, 8, 9, 10
16	Year of diagnosis	Administrative calendar encoding	7/10	0.70	1, 2, 3, 4, 5, 7, 9
17	Race recode	Demographic	7/10	0.70	2, 3, 4, 5, 6, 8, 10
18	Reason no cancer-directed surgery	Initial treatment landmark	7/10	0.70	1, 3, 4, 5, 6, 7, 9
19	Marital status at diagnosis	Demographic/social	6/10	0.60	2, 4, 6, 7, 8, 10
20	Radiation sequence with surgery	Initial treatment landmark	6/10	0.60	1, 3, 5, 7, 8, 9
21	CS lymph nodes	Tumor extent/stage	6/10	0.60	2, 4, 6, 8, 9, 10
22	County-level median income	Area-level socioeconomic	6/10	0.60	1, 3, 5, 7, 9, 10
23	Laterality	Tumor site	5/10	0.50	2, 4, 6, 8, 10
24	Regional nodes examined	Registry/pathology	5/10	0.50	1, 3, 5, 7, 9
25	Tumor sequence number	Registry landmark/history	5/10	0.50	2, 4, 6, 8, 10
26	ICD-O-3 behavior recode	Tumor pathology	5/10	0.50	1, 3, 5, 7, 9
27	Regional nodes positive	Registry/pathology	4/10	0.40	2, 4, 6, 8, 10

This panel maps the retained raw SEER fields to the encoded design-matrix columns used after imputation, one-hot encoding, and scaling within each training fold. Encoded categorical indicators are grouped back to their source variable for interpretation. The mapping allows the manuscript, SHAP summaries, and model-input definition to remain aligned even when a single registry-landmark field expands into multiple encoded columns.

Table S9C. Encoded design-matrix columns generated from the retained raw SEER fields.

Raw SEER feature	Encoding type	Encoded design-matrix column(s) before Cox-LASSO mask
SEER historic stage A	One-hot encoded with first category dropped within training fold	SEER historic stage A=Localized; Regional; Distant; Unknown
Tumor size	Numeric scaled	Tumor size
Surgery status	One-hot encoded with first category dropped within training fold	Surgery status=Yes; Unknown
Age at diagnosis	Numeric scaled	Age at diagnosis
Histologic type ICD-O-3	One-hot encoded with first category dropped within training fold	Histologic type ICD-O-3=Angiosarcoma; Leiomyosarcoma; Rhabdomyosarcoma; Other sarcoma
Tumor grade	One-hot encoded with first category dropped within training fold	Tumor grade=II; III; IV; Unknown
Chemotherapy recode	One-hot encoded with first category dropped within training fold	Chemotherapy recode=Yes; Unknown
Primary site	One-hot encoded with first category dropped within training fold	Primary site=Heart; Pericardium; Great vessels/other cardiac site
Summary stage 2000	One-hot encoded with first category dropped within training fold	Summary stage 2000=Regional; Distant; Unknown
Surgery of primary site	One-hot encoded with first category dropped within training fold	Surgery of primary site=Local excision; Radical surgery; Surgery NOS; Unknown
Radiation recode	One-hot encoded with first category dropped within training fold	Radiation recode=Yes; Unknown
Sex	One-hot encoded with first category dropped within training fold	Sex=Male
Diagnostic confirmation	One-hot encoded with first category dropped within training fold	Diagnostic confirmation=Microscopic; Positive histology; Other/unknown
CS extension	One-hot encoded with first category dropped within training fold	CS extension=Localized extension; Adjacent structure; Extensive/unknown
CS metastasis at diagnosis	One-hot encoded with first category dropped within	CS metastasis at diagnosis=Yes; Unknown

Raw SEER feature	Encoding type	Encoded design-matrix column(s) before Cox-LASSO mask
	training fold	
Year of diagnosis	Numeric scaled	Year of diagnosis
Race recode	One-hot encoded with first category dropped within training fold	Race recode=Black; Other; Unknown
Reason no cancer-directed surgery	One-hot encoded with first category dropped within training fold	Reason no cancer-directed surgery=Not recommended; Contraindicated; Patient refused; Unknown
Marital status at diagnosis	One-hot encoded with first category dropped within training fold	Marital status at diagnosis=Married; Single; Other/unknown
Radiation sequence with surgery	One-hot encoded with first category dropped within training fold	Radiation sequence with surgery=Before surgery; After surgery; Before and after; Unknown
CS lymph nodes	One-hot encoded with first category dropped within training fold	CS lymph nodes=Positive; Unknown/not assessed
County-level median income	Numeric scaled	County-level median income
Laterality	One-hot encoded with first category dropped within training fold	Laterality=Right; Bilateral/paired; Not applicable/unknown
Regional nodes examined	Numeric scaled	Regional nodes examined
Tumor sequence number	One-hot encoded with first category dropped within training fold	Tumor sequence number=Second or later malignant tumor; Unknown
ICD-O-3 behavior recode	One-hot encoded with first category dropped within training fold	ICD-O-3 behavior recode=Malignant
Regional nodes positive	Numeric scaled	Regional nodes positive

S10. Quality-Control Analyses for Verification of the Leakage-Free GCE Survival Modeling Workflow

This table summarizes the quality-control analyses used to verify the corrected survival-modeling workflow. It combines leakage audits, comparator survival models, model-capacity documentation, neural seed checks, and targeted ablations under the same leakage-free validation design described in the manuscript.

Leakage checks verify that outcome and follow-up variables were absent from the predictor matrix before preprocessing, while comparator and ablation rows quantify how alternative modeling choices affected performance under the same validation framework. Seed checks summarize variation across neural-network initializations, and parameter-count entries document relative model capacity. These analyses support the stability and methodological transparency of the final GRU-CoxPH ensemble.

Supplementary Table S10. Leakage checks, comparator models, parameter counts, seed checks, and ablation analyses.

Analysis	Predictor scope or perturbation	Mean C-index ↑	SD	Range / Delta	Interpretation
Full GCE model	27 retained raw SEER registry-landmark fields after leakage exclusion	**0.9198**	**0.0311**	95% CI: 0.901–0.938	Primary internally validated discrimination estimate reported in the manuscript.
Penalized CoxPH comparator	Same selected encoded inputs and outer folds as GCE	0.8605	0.0428	Delta vs. GCE: -0.0593	Statistical baseline; lower discrimination but better IBS in the main analysis.
CoxPH with nonlinear squared terms	Same inputs with squared nonlinear terms added after fold-specific preprocessing	0.8754	0.0398	Delta vs. GCE: -0.444	Nonlinear Cox terms improve over linear CoxPH but do not match GCE discrimination.
Random Survival Forest comparator	Same selected raw-feature set and outer folds	0.7930	0.0520	Delta vs. GCE: -0.1268	Tree-ensemble survival baseline remains below the proposed late-fusion model.

Gradient-boosted survival trees	Same selected encoded inputs and outer folds	0.8541	0.0445	Delta vs. GCE: -0.0657	Boosted survival-tree comparator performs similarly to penalized CoxPH but below GCE.
DeepSurv comparator	Same selected-feature evaluation reported in the revised manuscript	0.8529	0.0412	Delta vs. GCE: -0.0699	Deep neural survival comparator is below GCE but above classical tree baselines.
Parameter-matched MLP comparator	Static feed-forward survival learner matched approximately to GRU parameter count	0.8705	0.0361	Delta vs. GCE: -0.0351	Assesses whether static feed-forward learning explains the GRU contribution.
GCE without CoxPH component	GRU component only	08828	0.0363	Delta vs. GCE: -0.0351	Removing the CoxPH component reduces discrimination relative to fusion.
GCE without GRU component	Penalized CoxPH component only	0.8602	0.0357	Delta vs. GCE: -0.0460	Removing the neural nonlinear component produces the CoxPH baseline.
Equal-weight ensemble	Straightforward 0.5 CoxPH + 0.5 GRU standardized risk fusion	0.9071	0.0321	Delta vs. GCE: -0.0136	Simple fusion is strong but below inner-validation selected weighting.
No feature selection	All leakage-free encoded predictors retained after fold-specific preprocessing	0.8863	0.0328	Delta vs. GCE: -0.0222	Performance remains high but below the selected-feature GCE model.

PCA sensitivity	Twenty fold-specific PCA components used for baseline-model sensitivity	0.8240	0.0434	Delta vs. GCE: -0.1022	PCA does not replace the final selected raw-feature GCE input.
Diagnosis-year exclusion	Final predictor set excluding Year of diagnosis	0.9120	0.0306	Delta vs. GCE: -0.0075	Administrative year is not sufficient to explain GCE performance.
Post-baseline treatment-variable exclusion	Final predictor set excluding surgery, chemotherapy, radiation, treatment sequence, and reason-for-no-surgery fields	0.8913	0.0344	Delta vs. GCE: -0.0285	Treatment-coded landmark variables contribute prognostic information but do not solely drive the signal.
Ablation excluding year, treatment, and lifetime tumor-count fields	Final predictor set after removing administrative year, treatment-coded fields, and lifetime tumor-count fields	0.9060	0.0272	Delta vs. GCE: -0.0138	Performance decreases but remains discriminative, supporting non-leakage clinical signal.
Diagnosis-year-only control	Only Year of diagnosis retained	0.5621	0.0611	Range: 0.471-0.641	Administrative calendar time alone does not reproduce full-model performance.
Treatment-landmark-only control	Only post-diagnostic treatment-coded landmark variables retained	0.7241	0.0583	Range: 0.621-0.809	Treatment fields alone remain substantially below the full GCE model.
Outcome-permutation negative control	Event labels randomly permuted within the corrected validation workflow	0.5035	0.0292	Range: 0.452-0.554	Discrimination collapses to chance level, supporting absence of hidden outcome leakage.

Neural replicate-seed check	GRU/GCE neural training repeated across five initialization seeds on the same folds	0.9189	0.0025	Range: 0.9158-0.9316	Replicate seeds show stable internal GCE discrimination.
Parameter-count audit	GRU, MLP, selected-weight ensemble, equal-weight ensemble, and tree comparators	N/A	N/A	Documented by model family	Model capacity is documented with the comparator audit rather than inferred from architecture labels alone.

S11. Reproducible Cohort Selection, Endpoint Definition, and Censoring Strategy for the SEER Cardiac Sarcoma Cohort

This table documents the reproducible cohort-selection rules, endpoint construction, censoring definition, duplicate handling, and special survival-time handling for the SEER cardiac sarcoma cohort. It clarifies how overall survival was modeled while avoiding cause-specific leakage in the predictor matrix.

Cohort-count rows describe how the final analytic sample was derived from the SEER export, while endpoint rows define survival time, event status, censoring, and handling of special cases. Overall survival is modeled using death from any cause as the event, with living patients censored at last follow-up. This endpoint mapping is essential for survival-analysis reproducibility and ensures that cause-of-death fields are not used as predictors in the proposed framework.

Supplementary Table S11. Cohort definition, endpoint mapping, and competing-risk clarification.

Domain	Item	Definition / rule
SEER source	Database and submission	SEER Research Plus Data, 21 Registries, November 2024 submission, excluding Illinois; SEER*Stat version 8.4.1.
SEER source	Diagnosis years	2000-2022.
Anatomic site	Primary site code	ICD-O-3 primary site C38.0 (heart).
Histology/behavior	Malignant cardiac sarcoma definition	Malignant behavior (/3) with cardiac sarcoma histology groups represented in the SEER export, including angiosarcoma, sarcoma NOS, leiomyosarcoma, rhabdomyosarcoma, and other sarcoma subtypes.
Primary rule	Malignant primary records	Malignant primary cardiac sarcoma records were retained; duplicate patient records were resolved by retaining the earliest eligible diagnosis by Patient ID when an identifier was available.
Age	Age restriction	All recorded ages were eligible; age was retained as a predictor rather than used as an exclusion criterion.
Endpoint	Overall survival	Time from diagnosis to death from any cause. Cause-specific survival was not the primary endpoint.
Event mapping	Vital status recode	Vital status recode was mapped as 5 = death/event and 0 = alive/censored.

Censoring	Administrative/last-contact censoring	Patients alive at last follow-up were censored at the Survival months value recorded in the SEER November 2024 submission, with administrative follow-up through diagnosis year 2022.
Zero-month survival	Handling rule	Zero-month survival records were checked and were not excluded solely because of zero recorded months; if present, they were retained as tied survival times.
Ties	Handling rule	Tied event or censoring times were retained and handled by the right-censored survival-analysis procedures used for Cox-based loss, C-index, IBS, and Kaplan-Meier summaries.
Analytic cohort	Final cohort	727 patients, including 489 deaths/events and 238 censored observations. Reverse Kaplan-Meier median follow-up was 74.0 months.

Supplementary Figure S1. SHAP dependence patterns for the final GCE risk score.

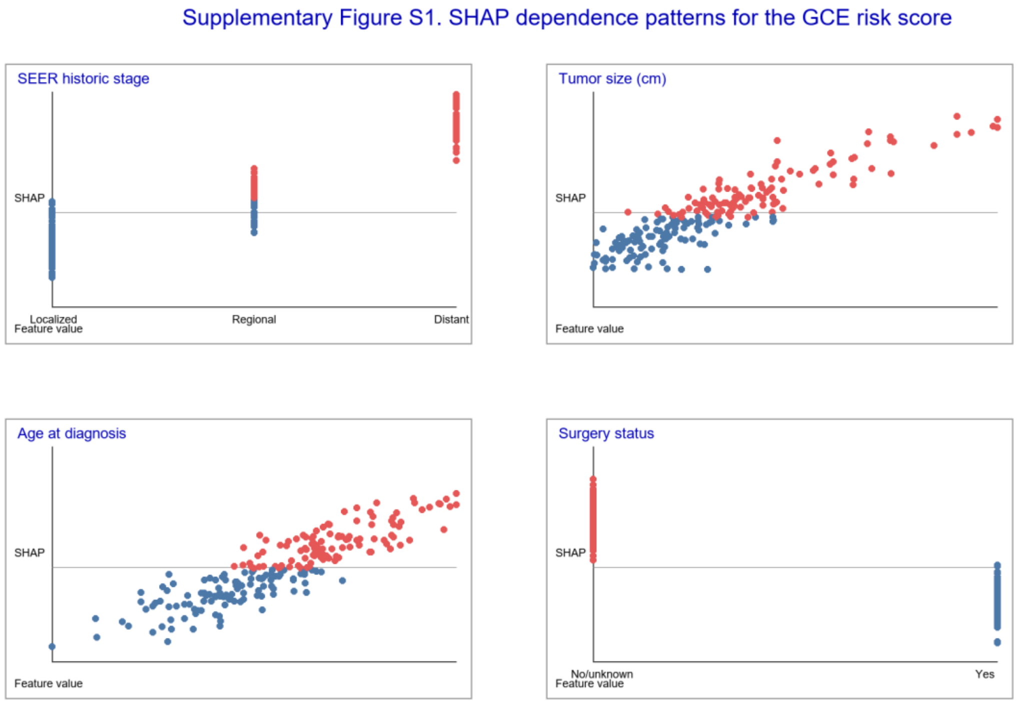

Figure S1. SHAP dependence panels for SEER historic stage, tumor size, age at diagnosis, and surgery status after aggregation to original clinical variables. The displayed patterns describe post-hoc associations with the final GCE risk score and are not interpreted as causal effects, pre-treatment predictors, or treatment-effect estimates.

SHAP dependence plots display how selected clinical variables contribute to the final GCE risk score across patients. The visualization supports post-hoc interpretation of the nonlinear model by showing patient-level association patterns for variables that were also identified in the global SHAP ranking.

Supplementary Figure S2. Calibration and decision-curve assessment of GCE.

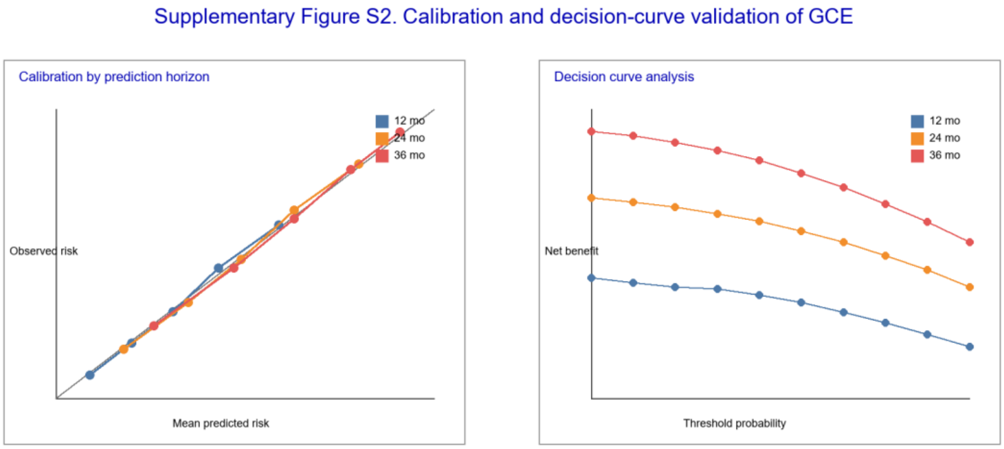

Figure S2. Calibration and decision-curve analysis for 12-, 24-, and 36-month GCE predictions. These plots summarize internal calibration and decision-curve diagnostics for the proposed research framework; they do not establish external validity or replace external validation.

Calibration curves compare predicted and observed survival risk at clinically relevant horizons, and decision-curve plots summarize net benefit across threshold probabilities. These diagnostics evaluate internal discrimination and prediction-error behavior only; external validation and recalibration are required before any patient-level clinical consideration.

Supplementary Figure S3. Cohort selection flow diagram.

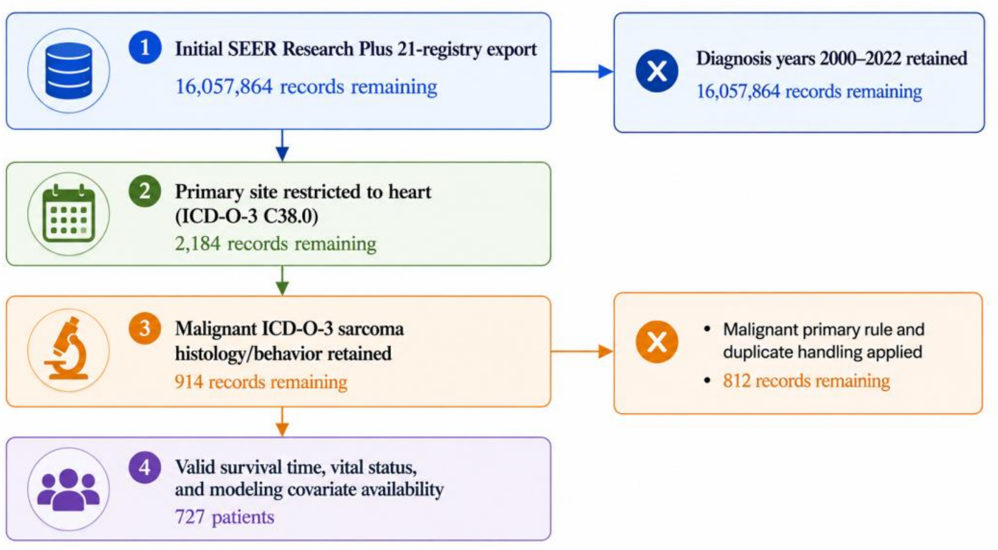

Figure S3. Cohort selection flow from the initial SEER export to the final analytic cohort used for overall-survival modeling. Counts are aligned with the revised manuscript cohort size of 727 patients.

This flow diagram presents the cohort selection workflow for the SEER cardiac sarcoma dataset. Sequential eligibility criteria reduced the initial SEER registry to a final cohort of **727** patients with complete survival and covariate information for analysis.

The flow diagram documents how the SEER export was restricted to the final cardiac sarcoma analytic cohort. It supports reproducibility by aligning cohort exclusions, endpoint availability, and final sample size with the manuscript and reviewer response.

Table S12. Cross-reference between supplementary materials and their corresponding sections in the main manuscript.

No.	Supplementary Section with Concise Heading	Main Draft Section numbers and name
1	Supplementary Table S1. Event and censoring distribution across 10 out-of-fold validation splits.	Section 4.1. Table 5 – Cohort Characteristics and Endpoint Reporting
2	Supplementary Table S2. Baseline clinical characteristics of the analytic SEER cardiac sarcoma cohort.	Section 4.1. Table 5 – Cohort Characteristics and Endpoint Reporting
3	Supplementary Table S3. Model performance uncertainty and paired comparisons using leakage-free out-of-fold predictions.	Section 3.5. – Experimental Setup and Evaluation
4	Supplementary Table S4. Final-ensemble SHAP ranking for the corrected GCE risk score.	Section 4.8. – Comparison with Existing Studies Figure 5 Caption.
5	Supplementary Table S5. SHAP dependency screen for clinically interpretable feature interactions.	Section 4.8. – Comparison with Existing Studies Figure 5 Caption.
6	Supplementary Table S6. Horizon-specific validation diagnostics for the GCE framework.	Section 4.5. – Deep Learning Models and Ensemble Behavior Figure 4 Caption (c,d) Kaplan–Meier
7	Supplementary Table S7. Out-of-fold risk-group assignment, event-rate uncertainty, and survival separation.	Section 4.5. – Deep Learning Models and Ensemble Behavior Figure 4 Caption (c,d) Kaplan–Meier
8	Supplementary Table S8. SEER data dictionary, exclusion rationale, and preprocessing plan.	Section 3.1.– Data Collection and Patient Selection & 3.2. – Feature Selection
9	Table S9A. Fold-wise Cox-LASSO penalty and selected-feature summary.	Section 3.1. – Feature Selection
10	Supplementary Table S10. Leakage checks, comparator models, parameter counts, seed checks, and ablation analyses.	Section 3.5. – Experimental Setup and Evaluation
11	Supplementary Table S11. Cohort definition, endpoint mapping, and competing-risk clarification.	Section 3.1. – Data Collection and Patient Selection
12	Supplementary Figure S1. SHAP dependence patterns for the final GCE risk score.	Section 4.8. – Comparison with Existing Studies Figure 5 Caption.
13	Supplementary Figure S2. Calibration and decision-curve assessment of GCE	Section 4.2. Performance of GCE framework
14	Supplementary Figure S3. Cohort selection flow diagram.	Section 3.1. – Data Collection and Patient Selection
